# A graph representation of functional diversity of brain regions

**DOI:** 10.1002/brb3.1358

**Published:** 2019-07-27

**Authors:** Dazhi Yin, Xiaoyu Chen, Kristina Zeljic, Yafeng Zhan, Xiangyu Shen, Gang Yan, Zheng Wang

**Affiliations:** ^1^ Institute of Neuroscience, Key Laboratory of Primate Neurobiology, CAS Center for Excellence in Brain Science and Intelligence Technology, Shanghai Institutes for Biological Sciences Chinese Academy of Sciences Shanghai China; ^2^ University of Chinese Academy of Sciences Beijing China; ^3^ School of Physics Science and Engineering Tongji University Shanghai China; ^4^ Kunming Institute of Zoology Chinese Academy of Sciences Kunming China

**Keywords:** functional brain networks, functional diversity, graph theory, human intelligence, neighbor dispersion index, resting‐state fMRI, topological architecture

## Abstract

**Introduction:**

Modern network science techniques are popularly used to characterize the functional organization of the brain. A major challenge in network neuroscience is to understand how functional characteristics and topological architecture are related in the brain. Previous task‐based functional neuroimaging studies have uncovered a core set of brain regions (e.g., frontal and parietal) supporting diverse cognitive tasks. However, the graph representation of functional diversity of brain regions remains to be understood.

**Methods:**

Here, we present a novel graph measure, the neighbor dispersion index, to test the hypothesis that the functional diversity of a brain region is embodied by the topological dissimilarity of its immediate neighbors in the large‐scale functional brain network.

**Results:**

We consistently identified in two independent and publicly accessible resting‐state functional magnetic resonance imaging datasets that brain regions in the frontoparietal and salience networks showed higher neighbor dispersion index, whereas those in the visual, auditory, and sensorimotor networks showed lower neighbor dispersion index. Moreover, we observed that human fluid intelligence was associated with the neighbor dispersion index of dorsolateral prefrontal cortex, while no such association for the other metrics commonly used for characterizing network hubs was noticed even with an uncorrected *p* < .05.

**Conclusions:**

This newly developed graph theoretical method offers fresh insight into the topological organization of functional brain networks and also sheds light on individual differences in human intelligence.

## 1 INTRODUCTION

A fundamental concern of cognitive neuroscience is how the functional organization of the human brain gives rise to adaptive behavior (Dehaene, Kerszberg, & Changeux, 1998; Miller & Cohen, 2001). Converging evidence from neuroimaging studies suggests that the human brain is configured by a core set of brain regions (e.g., frontal and parietal) supporting a wide variety of task demands (Duncan & Owen, 2000; Fedorenko, Duncan, & Kanwisher, 2013). Specifically, meta‐analyses of functional magnetic resonance imaging (fMRI) data have uncovered a common pattern of activity in response to many different kinds of cognitive tasks in frontoparietal cortex, extending to anterior insula, dorsal anterior cingulate cortex (dACC), and supplementary motor area (Niendam et al., 2012; Torta & Cauda, 2011; Yeo et al., 2015), areas that are collectively referred to as the multiple‐demand or cognitive control system (Dosenbach et al., 2007; Duncan, 2010). Therein, anterior insula and dACC are well‐known key nodes of the salience network (Ham, Leff, de Boissezon, Joffe, & Sharp, 2013; Seeley et al., 2007). Furthermore, high functional diversity in frontoparietal and anterior insular regions has been quantified based on the profiles of brain activation in terms of task domains involved (Anderson, Kinnison, & Pessoa, 2013). Beyond focal brain activation, a study using multitask functional connectivity analysis indicated that frontoparietal regions can flexibly and adaptively update their pattern of global connectivity to support implementation of multiple and varied tasks (Cole et al., 2013). Through dynamic network approaches, frontal‐related brain networks show flexible reconfiguration during effortful working memory in humans (Braun et al., 2015). These findings demonstrate that the frontoparietal cortices as well as brain regions comprising the salience network show high diversity in function, in contrast to functionally specialized regions such as primary sensorimotor areas.

Despite the benefits of aggregating data from task‐based neuroimaging studies in allowing us to characterize the functional diversity across brain regions (Genon, Reid, Langner, Amunts, & Eickhoff, 2018; Laird, Lancaster, & Fox, 2005; Yarkoni, Poldrack, Nichols, Van Essen, & Wager, 2011), the complete functional repertoire of a brain region remains intangible. This is because a very limited range of behavioral conditions have been investigated, in contrast to the immense range of human behaviors that occur in real‐life scenarios. Thus, the functional characterization of brain regions may be biased according to the experimental tasks recruited. Moreover, the brain has been described as intrinsically active, rather than passively stimulus‐driven (Engel, Gerloff, Hilgetag, & Nolte, 2013). Accordingly, resting‐state fMRI is widely used to examine intrinsic brain activity (Biswal, Yetkin, Haughton, & Hyde, 1995; Fox & Raichle, 2007). In particular, recent evidence suggests that resting‐state functional connectivity (network topology) describes the routes of task‐evoked activity flow (Cole, Ito, Bassett, & Schultz, 2016; Ito et al., 2017). This intrinsic network organization may capture the essence of brain function (Raichle, 2015). However, it remains to be understood how the topological structure of intrinsic functional networks is associated with the functional diversity of brain regions.

Graph theoretical approaches have been successfully used to quantify the topological properties of complex brain networks (Bullmore & Sporns, 2009; Kaiser, 2011; Liao, Vasilakos, & He, 2017; Rubinov & Sporns, 2010; Yan et al., 2015). Specifically, hubs with particular importance in a brain network can be characterized by a series of graph metrics (van den Heuvel & Sporns, 2013b), such as degree (Buckner et al., 2009), betweenness centrality (Freeman, 1977; Liang, Zou, He, & Yang, 2013), and participation coefficient (Guimera & Nunes Amaral, 2005; Power, Schlaggar, Lessov‐Schlaggar, & Petersen, 2013). Based on these graph metrics, network nodes can be characterized as different types of hubs, such as connector and provincial hubs (Fornito, Zalesky, & Breakspear, 2015). For example, betweenness centrality and participant coefficient have been employed to estimate connector hubs (Cole, Ito, & Braver, 2015). In particular, the participant coefficient (or a similar concept) was recently used and interpreted as a measure of diversity (Bertolero, Yeo, Bassett, & D'Esposito, 2018; Bertolero, Yeo, & D'Esposito, 2017; Betzel, Medaglia, & Bassett, 2018; Schultz et al., 2019). However, its calculation depends on modular decomposition of the network (Guimera & Nunes Amaral, 2005). Moreover, the modular structure can differ dramatically across network densities (Cole et al., 2015; Power et al., 2011), likely resulting in altered community assignment of a node and thus potentially affecting the measurement of the participant coefficient. Although Bertolero and colleagues (Bertolero et al., 2017) have shown that the participant coefficient is very consistent across densities (from 0.05 to 0.17), a previous study (Klimm, Borge‐Holthoefer, Wessel, Kurths, & Zamora‐Lopez, 2014) has argued that the measurement of participant coefficient is also potentially influenced by the size of communities, because the contribution of a node to each community depends on the size of the community. In addition, the participation coefficient cannot distinguish two nodes whose links are all within the same module. A method to measure nodal diversity that does not depend on the community partition is desired.

We therefore developed a new graph measure, the neighbor dispersion index (NDI), to describe the functional diversity of a given node based on the topological dissimilarity of its immediate neighbors in the network. That is, a brain region with higher diversity in function will have neighbors more dissimilar in topology, which may provide more possibilities for information spreading. Importantly, the calculation of NDI does not require preassumptions or depend on any other graph metrics (in contrast to the measurement of participant coefficient that requires a decomposition of functional network into modules). For an intuitive illustration, we first compared the NDI with several existing graph metrics (e.g., degree, betweenness centrality, and participant coefficient) commonly used for characterizing network hubs, in a hypothetical network. To test the ability and uniqueness of NDI in describing the functional diversity of brain regions, we subsequently applied the NDI and those hub‐related metrics in empirical resting‐state functional brain networks constructed from two independent and publicly accessible datasets. We hypothesized that functionally diverse regions such as in frontoparietal network and salience network would exhibit higher NDI compared with those functionally specialized areas such as in primary visual and sensorimotor networks.

In addition, human intelligence is a common factor that influences performance in a wide range of cognitive ability (Spearman, 1904). Accumulating neuroimaging studies suggest that human intelligence is linked to broad cognitive control functions of the frontal and parietal cortex (Duncan, 2010; Jung & Haier, 2007; Woolgar et al., 2010). Recent resting‐state fMRI studies revealed that frontoparietal connectivity profiles are predictive of human intelligence (Finn et al., 2015; Hearne, Mattingley, & Cocchi, 2016). To further validate the unique information captured by the NDI, we finally tested the association of each graph metric with individual differences in human intelligent behavior. We hypothesized that NDI values of frontal and parietal regions would be related to human intelligence.

## 2 MATERIALS AND METHODS

### 2.1 MRI datasets

Our analysis was performed on a resting‐state fMRI dataset of 100 unrelated participants (age: 29.1 ± 3.7 years; 46 male) from the Human Connectome Project (HCP), which exclude family relations and represent a sample of the general population. This dataset was collected through the [Link] (Van Essen et al., 2013). We also tested another resting‐state fMRI dataset of 198 subjects (Beijing site; age: 21.2 ± 1.8 years; 76 male) from the “1000 [Link] (FCP)” (Biswal et al., 2010). Data collection was approved by the institutional review board of the individual site, and informed consent was obtained from each subject.

### 2.2 Cognitive measures

Several cognitive measures were retrieved from the HCP database. We used the data from the NIH Toolbox behavioral measures, including Penn's Progressive Matrices, Picture Sequence Memory Test, Dimensional Change Card Sort Test, Picture Vocabulary Test, Pattern Comparison Processing Speed Test, and List Sorting Working Memory Test. For all measures, we used the raw, unadjusted values and the raw number of correct responses for Penn's Progressive Matrices following previous research (Schultz & Cole, 2016). There were no cognitive measures for the FCP database. The relationship between graph metrics and cognition could therefore not be tested in the FCP dataset.

### 2.3 Imaging acquisition

For the HCP dataset, the whole‐brain resting‐state fMRI scans were acquired with a 32 channel head coil on a modified 3 T Siemens Skyra. The sequence parameters are as follows: repetition time = 720 ms, echo time = 33.1 ms, flip angle = 52°, bandwidth = 2,290 Hz/Px, field of view = 208 × 180 mm, 72 slices, 2.0 mm isotropic voxels, with a multiband acceleration factor of 8 (Ugurbil et al., 2013). This dataset was collected across 2 days. On each day, 28 min of rest (eyes open with fixation) fMRI data were collected across two runs. In our current study, we only used the first run for each subject with 1,200 volumes. Details regarding the resting‐state data collection for this dataset have previously been described (Glasser et al., 2013). For the FCP dataset, scanning was performed on a 3 T MRI Scanner (Siemens). The whole‐brain resting‐state fMRI scans were acquired using an echo planar imaging sequence with repetition time = 2,000 ms, slices = 33, volumes = 225 (Liu & Duyn, 2013).

### 2.4 Resting‐state fMRI data preprocessing

For the HCP dataset, we used a minimally preprocessed volume version of the data, which underwent standard procedures including spatial normalization to a standard template, motion correction, and intensity normalization (Glasser et al., 2013); one subject was excluded due to poor image quality in the orbitofrontal cortex. We performed further preprocessing using SPM (SPM8; http://www.fil.ion.ucl.ac.uk/spm) and REST (Song et al., 2011) software. The first 10 volumes were discarded for signal equilibrium and to allow participants' adaptation to the scanning environment. We removed the variables of no interest from the time series using linear regression, including motion estimates, cerebrospinal fluid and white matter signals, and derivatives. In addition, we also conducted our analysis with global signal regression (GSR), given ongoing debate on this preprocessing step (Fox, Zhang, Snyder, & Raichle, 2009; Murphy, Birn, Handwerker, Jones, & Bandettini, 2009). The linear trend was removed, and the data were temporally bandpass filtered (0.01–0.08 Hz) and spatially smoothed (full width at half maximum = 4 mm).

For the FCP dataset, preprocessing was performed following our previous study (Yin et al., 2016), including slice timing, motion correction, spatial normalization (resampled to 3 mm isotropic voxels), smoothing (full width at half maximum = 8 mm), removing linear trends, temporal bandpass filtering (0.01–0.08 Hz), and regressing out covariates (i.e., six head motion parameters, cerebrospinal fluid, and white matter signals) (six subjects were excluded according to the notes in the database).

### 2.5 Construction of functional brain networks

We constructed functional brain networks based on preprocessed resting‐state fMRI images. For node definition, we used a parcellation scheme composed of 264 putative functional areas (Power‐264, spherical regions of interest (ROIs) with the same size; Power et al., 2011), as well as 90 ROIs according to the commonly used Automated Anatomical Labeling template (AAL‐90, ROIs with different size; Tzourio‐Mazoyer et al., 2002). The time series of each ROI was obtained by averaging the residual time courses of all voxels within the ROI. A symmetric *N* × *N* (*N* = 264 for the Power‐264 parcellation; *N* = 90 for the AAL‐90 template) functional connectivity matrix was generated, and elements of the matrix *A_ij_* represent the Pearson's correlation coefficient between the time courses of two ROIs, *i* and *j*. Functional connectivity matrices were thus obtained for each participant. The next step is selection of cost threshold (reflecting network density). The cost of the network *G* was defined as the total number of edges in a graph, divided by the maximum possible number of edges:

$$\cos t=\frac{1}{N\left(N-1\right)}\sum_{i\in G}K_{i},$$

where *K_i_* is the degree of node *i*, defined as the number of direct neighbors of a node.

However, there is currently no definitive criterion for threshold selection (Fornito, Zalesky, & Breakspear, 2013). Low cost may lead to a fragmented network with long shortest pathway, while high cost may reduce the economy of network. Considering that the network organization of the human brain is economic with features of small‐worldness and connectedness (Bassett & Bullmore, 2006; Bullmore & Sporns, 2009, 2012), we first calculated these topological properties of the functional brain networks across a wide range of cost (0.05 ≤ cost ≤ 0.5, with an incremental interval of 0.05) using graph theoretical analysis (see Methods S1 for the details of calculation of small‐worldness). We then selected the cost threshold that enables the brain network to be small‐world. In addition, we considered that the minimum mean degree of nodes across subjects should be >1, to minimize the disconnected nodes. We finally validated our main findings across cost thresholds within small‐world regime.

### 2.6 Neighbor dispersion index of each node in a network

**Figure 1:**
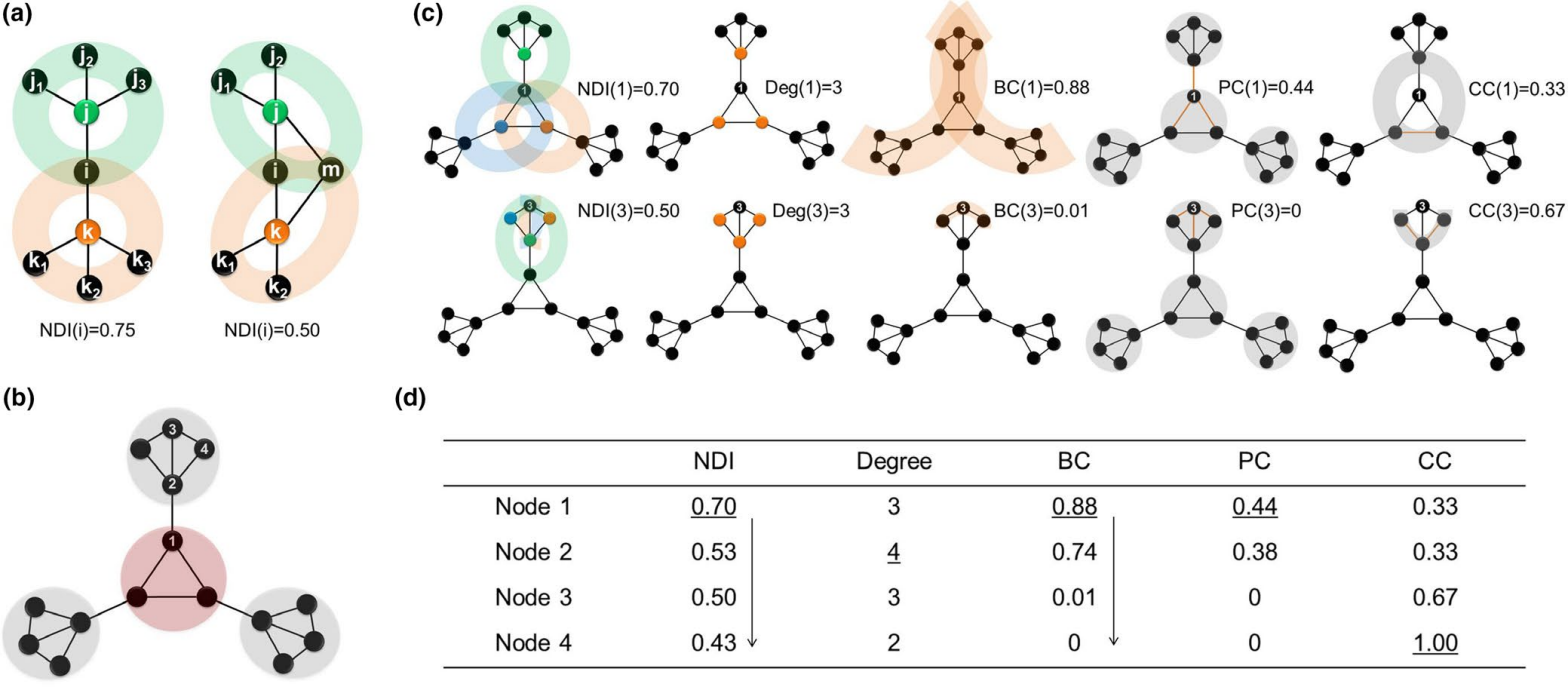
Illustration of the concept and performance of our proposed graph metric NDI in hypothetical networks. (a) A toy graph shows NDI of node *i*, in which *j* and *k* are neighbors of *i*. The colored shadows indicate neighbors' connectivity patterns of node *i* (the color corresponds to that of the neighbor node). The smaller the overlap among nodes in the neighbors' connectivity patterns, the higher the NDI of node *i*. (b) A hypothetical network is composed of a core and integrated system and three peripheral and segregated systems. Four nodes likely having different topological roles are labeled by numbers. (c) A graph illustration of the two nodes (i.e., node 1 and 3) with the same degree (*K* = 3) but in different systems for each metric. The colored shadows, nodes, and edges represent the topological information captured by each graph metric. (d) Comparison between NDI and four commonly used graph metrics for the four nodes. The maximum value of each metric among the four nodes was labeled with underlines. The arrows indicate the values following a descending order, suggesting the ability of those metrics to distinguish the topological role of different nodes. BC, betweenness centrality; CC, clustering coefficient; NDI, neighbor dispersion index; PC, participation coefficient

Here, we assume that the functional diversity of a brain region is embodied by topological differentiation of its immediate neighbors in the large‐scale functional brain network. To characterize the functional diversity of each node in a network, we propose a new graph metric NDI, quantified by the ratio of topological distance between immediate neighbors of a given node *i* and the maximum possible distance:

$$\text{NDI}\left(i\right)=\frac{\sum_{s,t\in G_{i}}d_{\mathrm{st}}}{\left(K_{i}-1\right)\times \sum_{j\in G_{i}}K_{j}},$$

where *G_i_* denotes the set of nodes that are the immediate neighbors of the node *i*, *K_i_* is the degree of *i*, *d_st_* indicates topological distance between two nodes, *s* and *t*, defined as:

$$d_{\mathrm{st}}=\sum_{m=1}^{N}\left|A_{\mathrm{sm}}-A_{\mathrm{tm}}\right|,$$

where *A* is the adjacency matrix, here the distance is evaluated using Manhattan distance. The NDI of a node is close to 1 if the neighbors of its neighbors are largely nonoverlapped and 0 if the neighbors of its neighbors are completely overlapped or its degree is <2. The NDI measures the degree of dispersion of information from a node in the network. To illustrate the concept of NDI, a toy graph is shown in Figure 1a.

### 2.7 Existing graph metrics commonly used for identifying network hubs

For comparison purposes, we calculated three existing graph metrics, commonly used for identifying network hubs, including degree, betweenness centrality, and participation coefficient. Degree *K_i_* is defined as the number of immediate neighbors of a node *i*. Betweenness centrality *B_i_* is defined as the number of shortest paths between any two nodes that run through node *i* (Freeman, 1977):

$$B_{i}=\sum_{j,k\in N,j\neq k}\frac{n_{j,k}\left(i\right)}{n_{j,k}},$$

where *n_j,k_* (*i*) is the number of shortest paths between nodes *j* and *k* that run through node *i* and n*_j,k_* is all shortest paths between nodes *j* and *k*. *B_i_* captures the influence of a node over information flow between other nodes in the network, which tends to be high for brain regions with extensive across‐network connectivity. The participation coefficient *P_i_* of node *i* is defined as follows (Guimera & Nunes Amaral, 2005):

$$P_{i}=1-\sum_{s=1}^{N_{M}}{\left(\frac{K_{\mathrm{is}}}{K_{i}}\right)}^{2},$$

where *N_M_* is number of modules, identified by a spectral optimization algorithm (Newman, 2006), *K_is_* is the number of links of node *i* to nodes in module *s*, and *K_i_* is the degree of node *i*. The participation coefficient of a node is close to 1 if its links are uniformly distributed among all the modules and 0 if all its links are within its own module.

To consider that the contribution of a node to each community also depends on the size of the community, Klimm and colleagues have introduced refined versions of the participation coefficient, called the participation index (*pi*) and the dispersion index (*di*; Klimm et al., 2014). We also calculated these two indices as a comparison. The formulas of these two indices are as follows:

$$pi=1-\frac{N_{M}}{\sqrt{N_{M}-1}}\sigma \left(PV_{i}\right),$$

where *N_M_* indicates the number of modules of a given network, *σ*(·) evaluates the standard deviation of the elements, *PV_i_* denotes participation vector whose elements *PV_is_* represent the probability that node *i* belongs to module *s*, *s* = 1, 2, 3, …, *N_M_*, the probability is given by *PV_is_* = *K_is_*/*N*, where *K_im_* is the number of links of node *i* to nodes in module *s*, *N* is the size of the module. To eliminate the effect of the degree *K_i_*, here the participation vector *PV_i_* is normalized such that $\sum_{s=1}^{N_{M}}PV_{\mathrm{is}}=1$.

The *pi* reflects the global reach of a node's links among all the modules. In contrast, the *di* is proposed as a measure of how difficult it is to classify a node into only one module. The definition of *di* is equivalent to the *pi* but considering only the nonzero entries of the participation vector:

$$di=1-\frac{N_{M}^{\prime }}{\sqrt{N_{M}^{\prime }-1}}\sigma \left(PV_{i}^{\prime }\right),$$

where $PV_{i}^{\prime }$ is the subvector containing only the nonzero entries of *PV_i_*, and $N_{M}^{\prime }$ is the dimension of $PV_{i}^{\prime }$.

### 2.8 Correlation analysis between each metric and human intelligence across the whole brain

We used Penn's Progressive Matrices as a measure of fluid intelligence (Bilker et al., 2012; Prabhakaran, Smith, Desmond, Glover, & Gabrieli, 1997). General intelligence, composed of fluid intelligence (novel/flexible processing; e.g., solving a novel problem) and crystallized intelligence (learned/stereotyped processing; e.g., vocabulary knowledge), is a broader construct than fluid intelligence (Horn & Cattell, 1966). We estimated general intelligence by considering scores on multiple measures of cognition function using factor analysis. We included scores from tests of fluid intelligence (Penn's Progressive Matrices), episodic memory (Picture Sequence Memory Test), executive function and cognitive flexibility (Dimensional Change Card Sort Test), language and vocabulary comprehension (Picture Vocabulary Test), processing speed (Pattern Comparison Processing Speed Test), and working memory (List Sorting Working Memory Test). We aggregated these six scores using principal component analysis and treated the first component as an evaluation of general intelligence (Schultz & Cole, 2016). We performed correlation analyses between all brain regions (i.e., AAL‐90 atlas) and each measure of intelligence (i.e., fluid intelligence and general intelligence), resulting in 90 Pearson correlation coefficients. For each correlation coefficient, a permutation test where the intelligence scores of individuals were shuffled 5,000 times yielded a nonparametric *p*‐value. Nonparametric *p*‐values for all brain regions were corrected for multiple comparisons (i.e., 90) using the Bonferroni method (a corrected cutoff *p*‐value = .05/90 = .00056). Finally, both corrected and uncorrected *p*‐values combined with observed correlation coefficients were reported. To validate the association between NDI and human intelligence, we further conducted the above correlation analysis across network densities, preprocessing strategies (without/with GSR), and brain parcellations. The validation analyses were not independent of the primary tests and were therefore not corrected.

### 2.9 Statistical analysis

To show the brain map of each graph metric, we calculated the mean value of each brain region across all participants. In order to quantify whether and how values of each metric varied across brain regions and potential functional roles, we divided the brain into 10 different functional networks according to a previous study (Power et al., 2011). Then, a one‐way analysis of variance (ANOVA) was performed on the mean values of each metric within these functional networks. Finally, post hoc two‐sample *t*‐tests were conducted to assess the significant differences in the mean values of each metric between any two functional networks. A threshold of *p* < .05 with Bonferroni correction was considered statistically significant. To identify distinguishability of NDI, we performed Pearson correlation analyses between NDI and the other metrics across the whole brain, and *r* values are reported.

## 3 RESULTS

### 3.1 Comparing NDI with several commonly used graph metrics for identifying network hubs in a hypothetical network

We first illustrated the performance of our proposed graph metric NDI by comparing it with four commonly used graph metrics, that is, degree, betweenness centrality, participation coefficient, and clustering coefficient, in a hypothetical network (Figure 1b). The hypothetical network is composed of a core, integrated system and three peripheral, segregated systems. Four nodes likely having different topological roles are labeled numerically. Figure 1c shows the topology patterns of the neighbors of two nodes with the same degree (*K* = 3) but in different systems as well as graph illustration for other metrics. The NDI of the node in the core system is greater than that of the node in the peripheral system (0.7 vs. 0.5). The complete comparison shows that NDI and betweenness centrality can consistently distinguish between the four nodes, but degree, participation coefficient, and clustering coefficient cannot (Figure 1d). This result suggests that NDI and betweenness centrality are comparable in characterizing the diversity of nodes for the hypothetical network.

### 3.2 Comparing NDI with several commonly used graph metrics for identifying network hubs in empirical resting‐state functional brain networks

**Figure 2:**
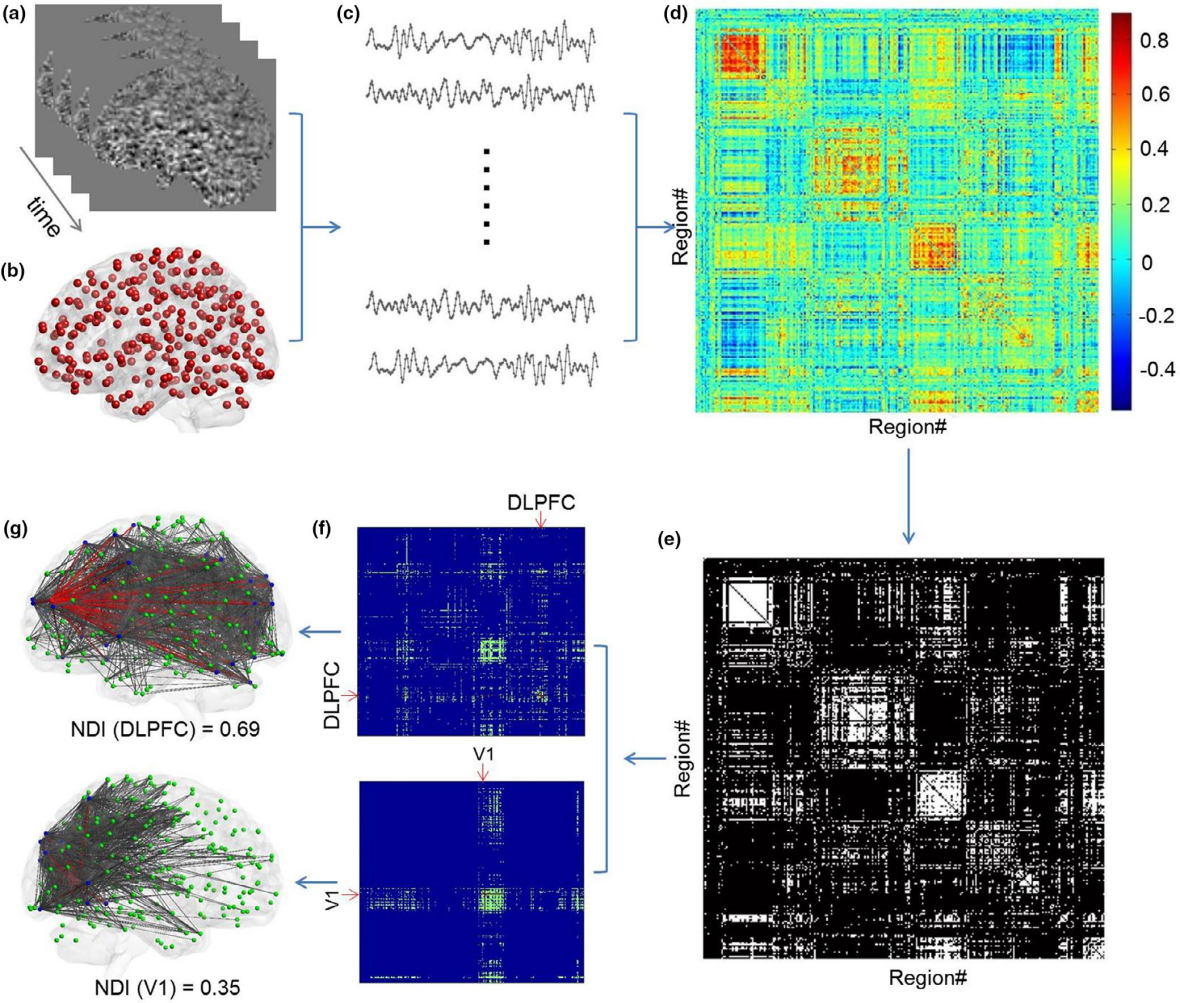
Illustration of the procedures for calculating the NDI in a single subject (HCP#100307). Based on preprocessed functional images (a), mean time course of each region of interest (here using Power‐264 parcellation (b)) was extracted (c). Pearson correlation analysis was then performed for the time courses of each pair of brain regions, and thus, a functional connectivity matrix was obtained (the size of matrix is 264 × 264) (d). To construct a functional brain network, network sparsification using a density threshold is often needed (here cost = 0.15) (e). Finally, the NDI of each brain region was calculated on the basis of topological dissimilarity of its immediate neighbors. (f) shows the connectivity patterns of two representative nodes (i.e., DLPFC and V1) and their immediate neighbors. The topological architecture of immediate neighbors (blue nodes) for the two representative nodes (red nodes) is also shown, respectively (g). The connectivity pattern (gray lines) of DLPFC's immediate neighbors is dispersed and almost covers the whole brain. In contrast, the connectivity pattern (gray lines) of V1's immediate neighbors is localized and primarily concentrated on visual cortex and posterior parietal cortex. The red lines denote direct connections between DLPFC/V1 and their immediate neighbors. DLPFC, dorsolateral prefrontal cortex; NDI, neighbor dispersion index; V1, primary visual cortex

We next applied NDI and commonly used graph metrics to the empirical functional brain networks constructed from the HCP dataset. The main results reported here are based on a single network density (cost = 0.15), which was identified under the consideration that network organization of the human brain is economic with features of small‐worldness and connectedness (Figure S1). Figure 2 illustrates the procedures for calculating the NDI in a single subject (HCP#100307). For intuitive understanding of the NDI, we depicted the topological architecture of the immediate neighbors for two representative nodes with NDI equal to 0.69 and 0.35, located in the dorsolateral prefrontal cortex (DLPFC) and primary visual cortex (V1), respectively. The connectivity pattern of DLPFC's immediate neighbors is dispersed and almost covers the whole brain. In contrast, the connectivity pattern of V1's immediate neighbors is localized, mainly concentrated in the visual cortex and posterior parietal cortex.

**Figure 3:**
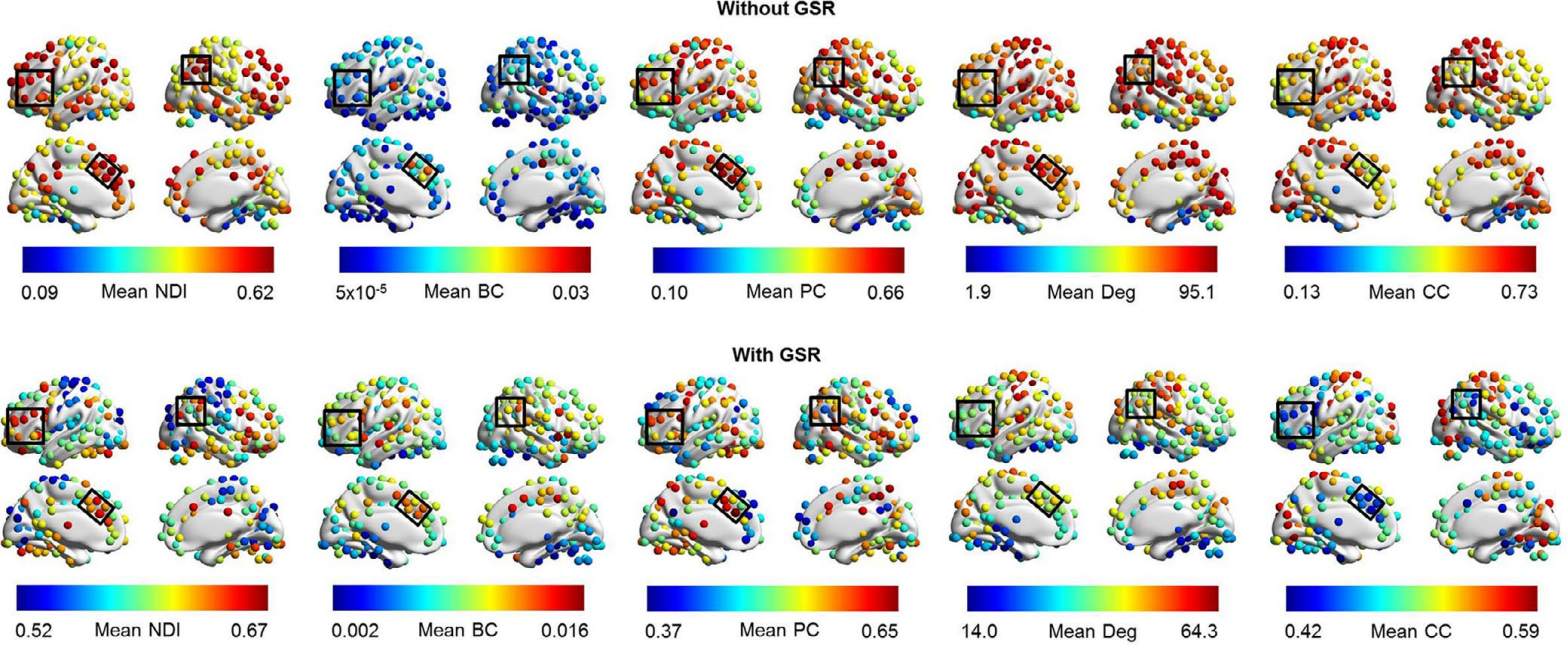
Mean values of each node across participants for both without and with GSR. The color bar denotes the magnitude of mean values. The black squares highlight the brain regions in the lateral prefrontal cortex, anterior insula, inferior parietal lobule, dorsal anterior cingulate cortex, and supplementary motor area. BC, betweenness centrality; CC, clustering coefficient; Deg, degree; GSR, global signal regression; NDI, neighbor dispersion index; PC, participation coefficient

At the nodal level, we found that lateral prefrontal cortex, inferior parietal lobule, anterior insula, dACC, and supplementary motor area consistently showed higher NDI regardless of data preprocessing without or with GSR, but not betweenness centrality, participant coefficient, degree, and clustering coefficient (Figure 3). Across the whole‐brain regions, we found weak correlations (without/with GSR) between NDI and betweenness centrality (*r* = .17/*r* = .11) and participant coefficient (*r* = 0/*r* = .31) and degree (*r* = −.44/*r* = −.49). Despite a relatively high (anti) correlation between NDI and clustering coefficient (*r* = −.75/*r* = −.89), the correlation is not perfect (Figure S2). For instance, many brain regions show clustering coefficients equal to 0 and 1. However, their NDI values are widely distributed. This is because the clustering coefficient takes into account only direct connections among immediate neighbors of a given node, while NDI captures information of entire topological patterns of the neighbors, which may explain the remaining variances. This result indicates that NDI is distinguishable from the other graph metrics and able to capture unique topological information.

**Figure 4:**
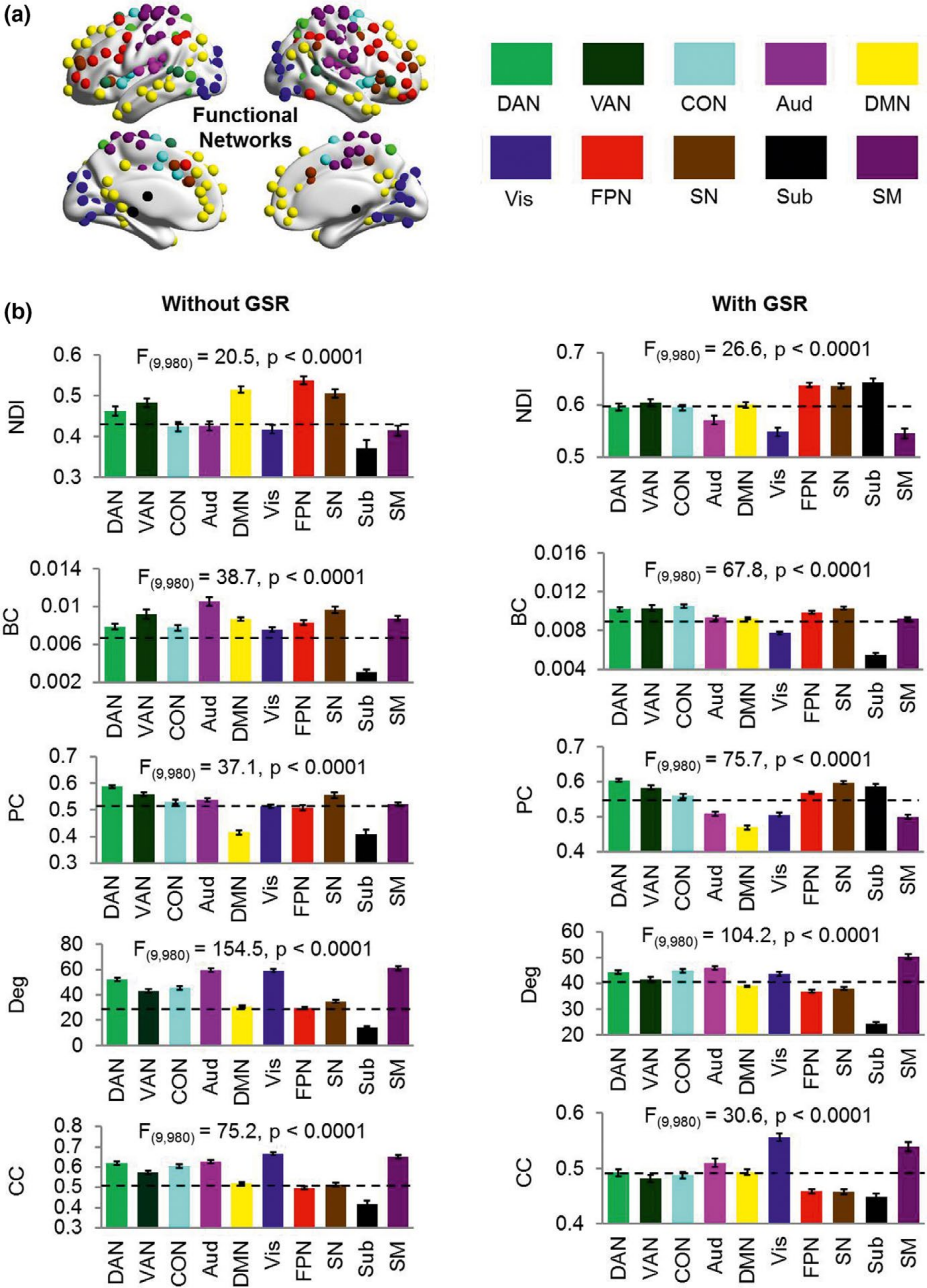
(a) Ten different functional networks of human brain were divided according to a previous study (Power et al., 2011). (b) Mean values of each functional network both without and with GSR. The mean values of each functional network were calculated across all participants and across regions within that functional network. Error bars stand for *SEM*. The dashed lines denote the global mean value across all functional networks. A one‐way analysis of variance (ANOVA) was performed on the mean values of each metric within these functional networks. Aud, auditory network; BC, betweenness centrality; CC, clustering coefficient; CON, cingulate‐opercular network; DAN, dorsal attention network; Deg, degree; DMN, default mode network; FPN, frontoparietal network; GSR, global signal regression; NDI, neighbor dispersion index; PC, participation coefficient; *SEM*, standard error of the mean; SM, sensorimotor network; SN, salience network; Sub, subcortical network; VAN, ventral attention network; Vis, visual network

At the network level, we consistently observed that the brain map of each metric was heterogeneous across functional networks (i.e., without GSR: ANOVA, *F*_(9,980)_ = 20.5, *p* < .0001 for NDI; *F*_(9,980)_ = 38.7, *p* < .0001 for betweenness centrality; *F*_(9,980)_ = 87.0, *p* < .0001 for participant coefficient; *F*_(9,980)_ = 154.5, *p* < .0001 for degree; *F*_(9,980)_ = 75.2, *p* < .0001 for clustering coefficient; and with GSR: ANOVA, *F*_(9,980)_ = 26.6, *p* < .0001 for NDI; *F*_(9,980)_ = 67.8, *p* < .0001 for betweenness centrality; *F*_(9,980)_ = 75.7, *p* < .0001 for participant coefficient; *F*_(9,980)_ = 104.2, *p* < .0001 for degree; *F*_(9,980)_ = 30.6, *p* < .0001 for clustering coefficient). Post hoc results (*p* < .05, Bonferroni corrected) showed that NDI of the frontoparietal network and the salience network was significantly higher than that of the auditory, visual, and sensorimotor networks for both without and with GSR conditions. However, this was not the case for betweenness centrality, participant coefficient, degree, or clustering coefficient. Specifically, auditory network and salience network showed higher betweenness centrality; dorsal attention network and salience network showed higher participant coefficient; auditory and sensorimotor networks showed higher degree; and visual and sensorimotor networks showed higher clustering coefficient (Figures 4 and S3). This result demonstrates that NDI is capable of describing functional diversity across human brain.

To show the uniqueness of NDI, we also calculated *pi* and *di*, as refined versions of the participation coefficient. Although the salience network showed higher *pi* and *di*, we found that the frontoparietal network did not show higher *pi* or *di* (Figure S4). This result not only suggests that the size of modules may not significantly affect the measure of participation of nodes in the empirical functional brain networks, but also further implies that our proposed NDI captures connectivity diversity in a manner that is different from graph metrics based on modular decomposition.

### 3.3 Validation analysis of the methodology

**Figure 5:**
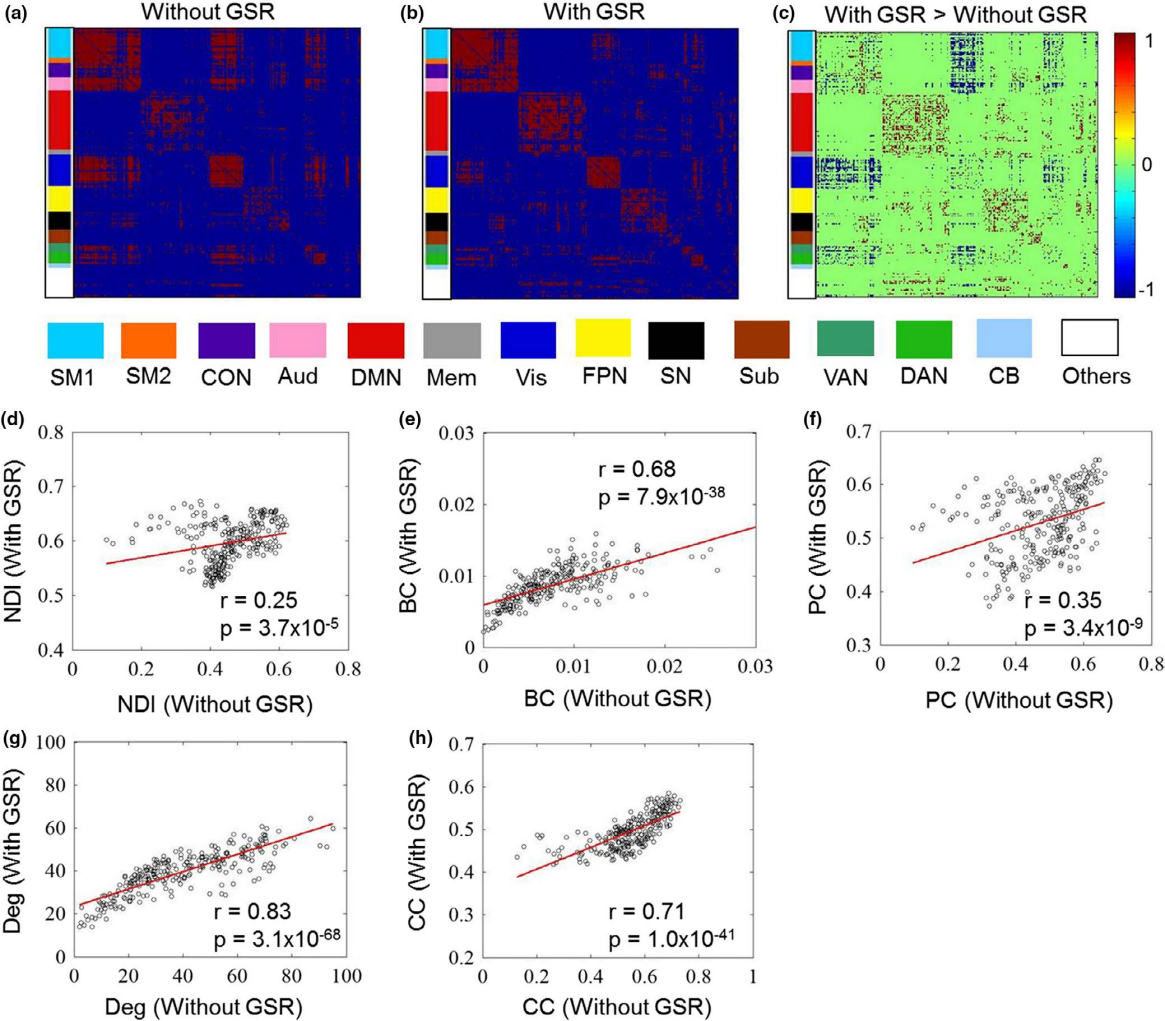
Functional connectivity matrices (cost = 0.15) without (a) and with (b) GSR, as well as the differences of the two conditions (c, with GSR > without GSR). Correlation of each graph metric between the two conditions was also shown (d–h). Aud, auditory network; BC, betweenness centrality; CB, cerebellum; CC, clustering coefficient; CON, cingulate‐opercular network; DAN, dorsal attention network; Deg, degree; DMN, default mode network; FPN, frontoparietal network; GSR, global signal regression; Mem, memory retrieval network; NDI, neighbor dispersion index; PC, participation coefficient; SM, sensorimotor network; SN, salience network; Sub, subcortical network; VAN, ventral attention network; Vis, visual network

To test the effect of GSR, we first showed the differences of functional connectivity matrices without and with GSR. We found that the differences were distributed across the whole matrix (Figure 5a–c). Through correlation analysis of each graph metric between conditions without and with GSR, we observed significant correlations for NDI (*r* = .25, *p* = 3.7 × 10^–5^), betweenness centrality (*r* = .68, *p* = 7.9 × 10^–38^), participant coefficient (*r* = .35, *p* = 3.4 × 10^–9^), degree (*r* = .83, *p* = 3.1 × 10^–68^), and clustering coefficient (*r* = .71, *p* = 1.0 × 10^–41^), although correlations for NDI and participant coefficient were relatively weaker (Figure 5d–h). This result suggests that NDI and participant coefficient are more sensitive to the changes of network topology due to GSR.

**Figure 6:**
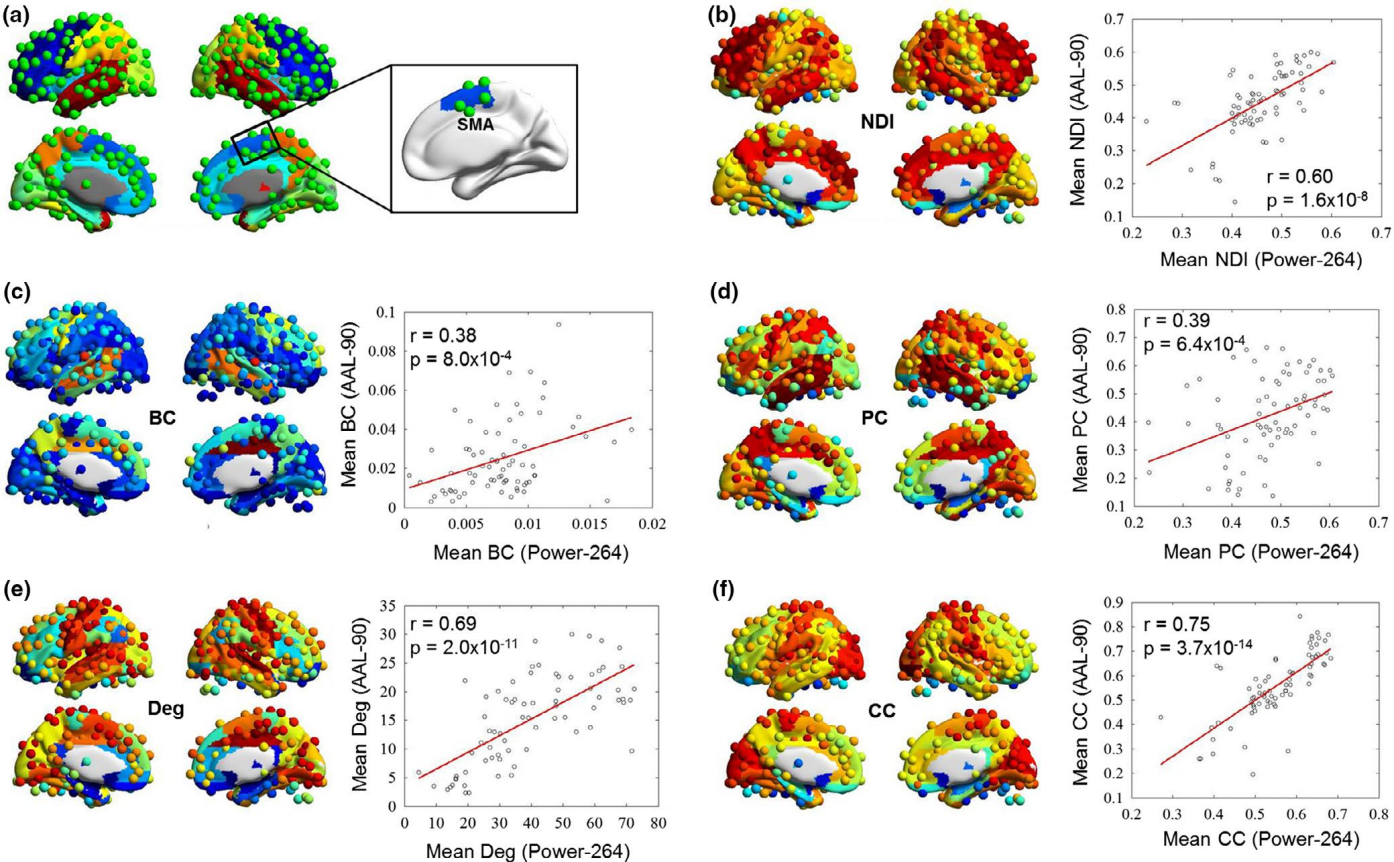
Robust brain map of each graph metric for different brain parcellations. An overlay of Power‐264 and AAL‐90 parcellations was shown in (a); however, only 73 AAL regions have overlapping spherical nodes in Power‐264. The mean values of spherical nodes within each AAL region were first calculated. Then, Pearson correlation analysis of each graph metric across brain regions was performed between the two brain parcellations (b–f). BC, betweenness centrality; CC, clustering coefficient; Deg, degree; NDI, neighbor dispersion index; PC, participation coefficient; SMA, supplementary motor area

To test the effect of different brain parcellations, we calculated the NDI and the other metrics based on another commonly used AAL‐90 template. We found that the brain maps of NDI (*r* = .60, *p* = 1.6 × 10^–8^), betweenness centrality (*r* = .38, *p* = 8.0 × 10^–4^), participant coefficient (*r* = .39, *p* = 6.4 × 10^–4^), degree (*r* = .69, *p* = 1.6 × 10^–11^), and clustering coefficient (*r* = .75, *p* = 3.7 × 10^–14^) were matched between the two brain parcellations (Figure 6). This result suggests that our findings are robust to different brain parcellations.

**Figure 7:**
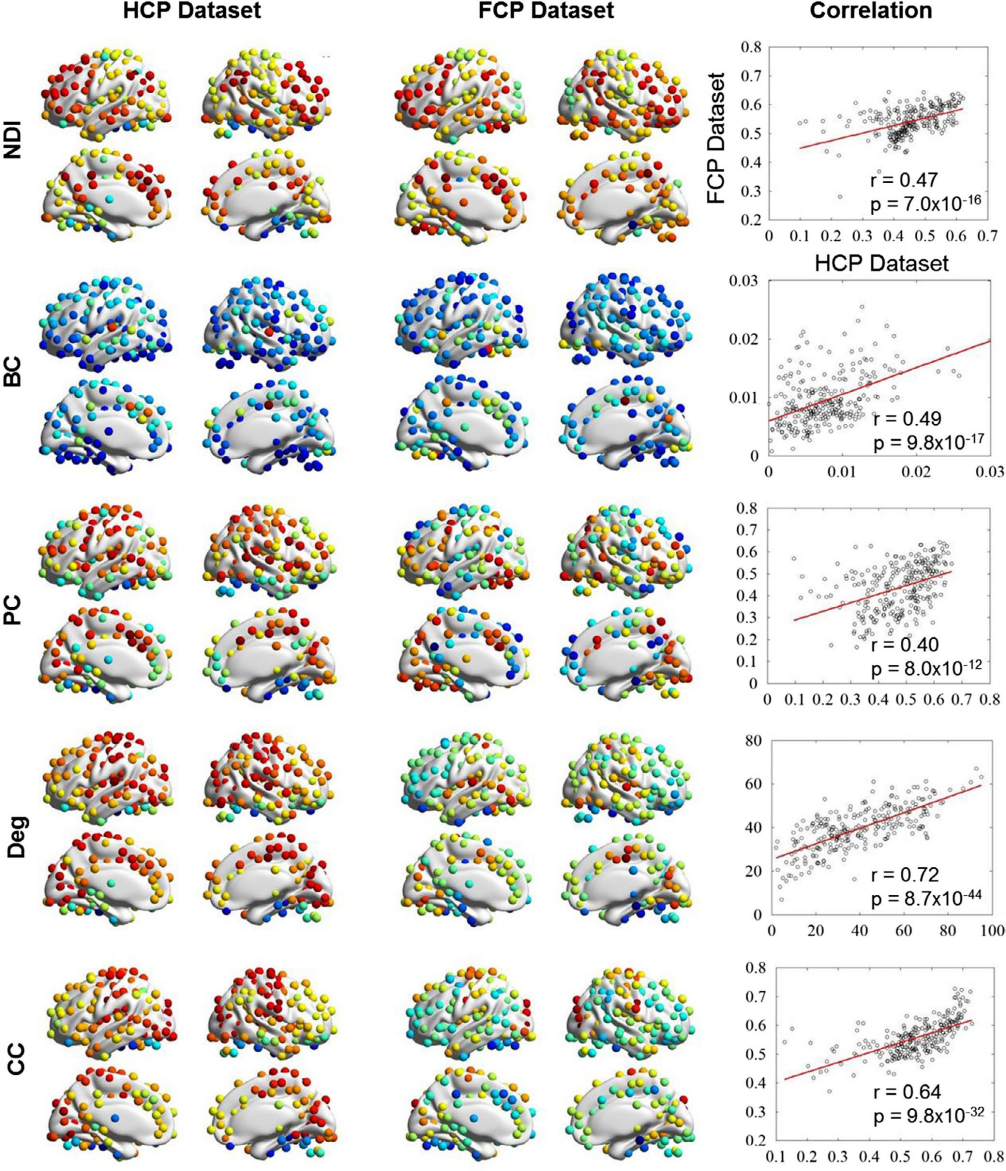
Robustness brain map of each graph metric for different resting‐state fMRI datasets. To reproduce the brain map of each graph metric in a second dataset, another 198‐subject resting‐state fMRI dataset from the database known as “1000‐FCP” was also analyzed. Significant correlation of each graph metric across brain regions was found between the HCP and FCP datasets. BC, betweenness centrality; CC, clustering coefficient; Deg, degree; FCP, Functional Connectomes Project; fMRI, functional magnetic resonance imaging; HCP, Human Connectome Project; NDI, neighbor dispersion index; PC, participation coefficient

To reproduce the brain map of each graph metric in a second dataset, we analyzed a 198‐subject resting‐state fMRI dataset from the database known as “1000‐FCP.” We found that the brain maps of NDI (*r* = .47, *p* = 7.0 × 10^–16^), betweenness centrality (*r* = .49, *p* = 9.8 × 10^–17^), participant coefficient (*r* = .40, *p* = 8.0 × 10^–12^), degree (*r* = .72, *p* = 8.7 × 10^–44^), and clustering coefficient (*r* = .64, *p* = 9.8 × 10^–32^) were consistent between the two datasets (Figure 7). This result implies that the brain map of each metric is robust to different fMRI datasets even with different temporal and spatial resolutions, and pipelines of data preprocessing.

**Figure 8:**
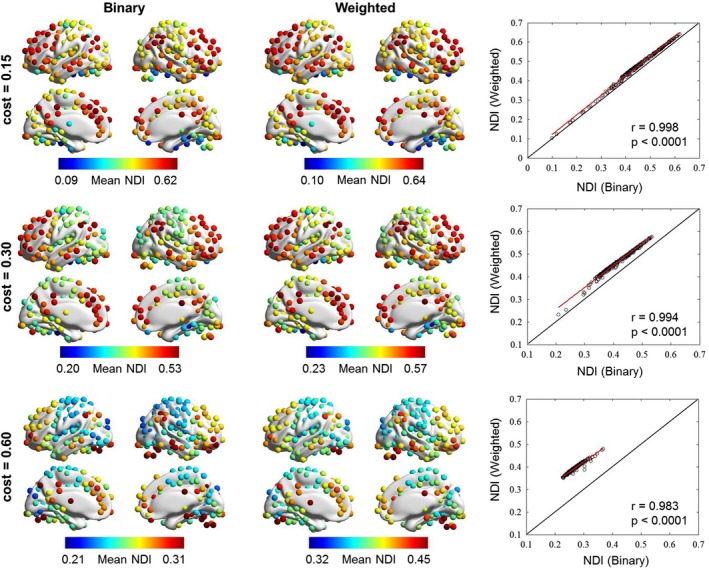
Applicability of NDI in both binary and weighted networks with different densities (i.e., cost = 0.15, 0.30, and 0.60). The regional profile of NDI is highly consistent between the binary and weighted networks across a wide range of densities. However, the quantitative differences of NDI between binary and weighted networks are dramatic at higher network density (cost = 0.60). NDI, neighbor dispersion index

In the above analyses, the values of NDI were calculated for binary networks. To test the applicability of NDI in networks with connectivity weights, we applied it to weighted functional brain networks with a wide range of network densities (i.e., cost = 0.15, 0.30, and 0.60), without setting a density threshold (however, all negative correlations were set to zero as NDI is not applicable for negative weights). We found the regional profile of NDI to be highly consistent between the binary and weighted networks (*r* = .998, *p* < .0001 for cost = 0.15; *r* = .994, *p* < .0001 for cost = 0.30; and *r* = .983, *p* < .0001 for cost = 0.60). However, the quantitative differences in NDI between binary and weighted networks are dramatic at a higher network density (cost = 0.60; Figure 8).

**Figure 9:**
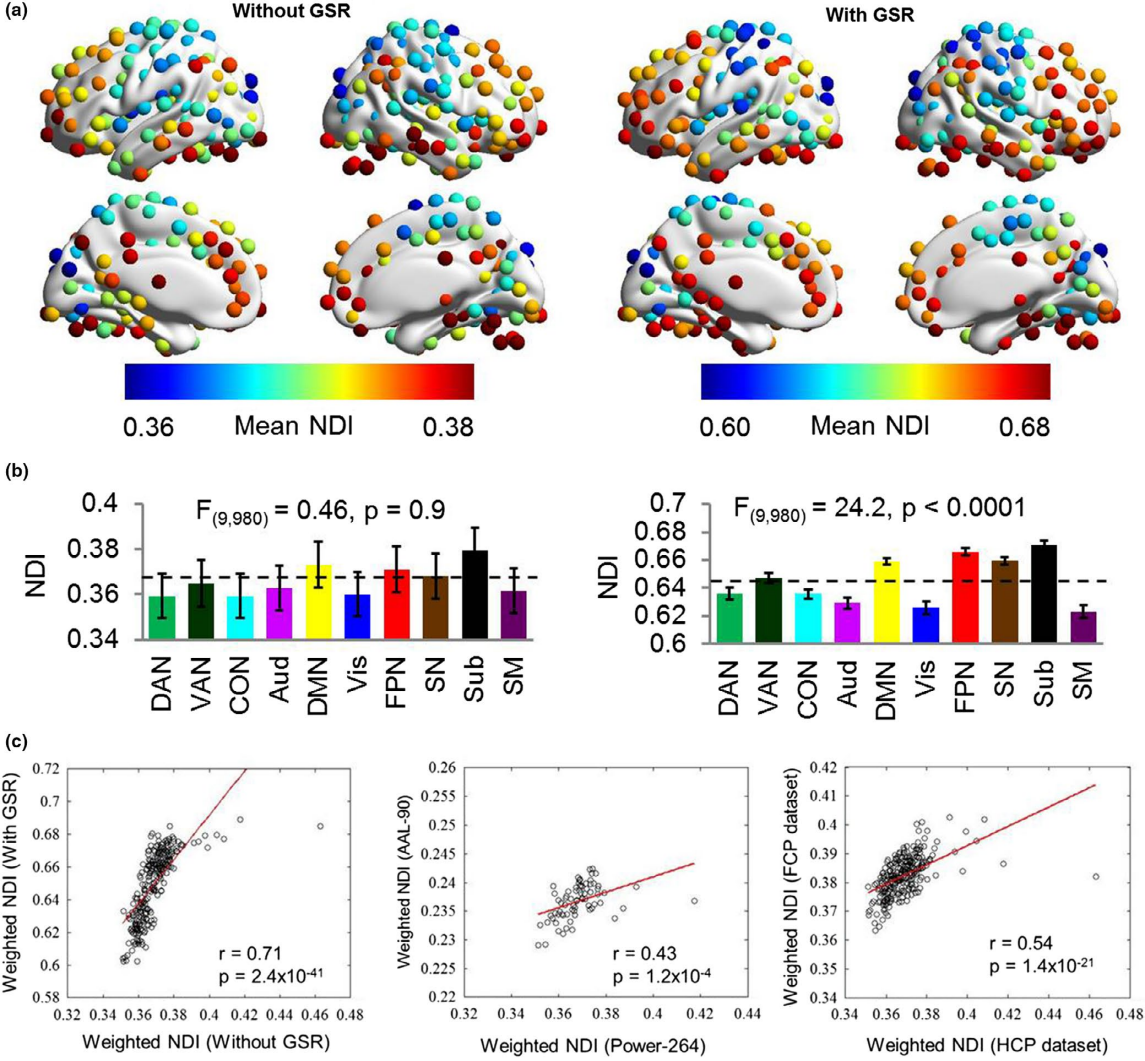
Applicability of NDI in weighted networks without setting a density threshold. (a) Mean NDI values of each node across participants for both without and with GSR. The color bar denotes the magnitude of mean values. (b) Mean NDI values of each functional network both without and with GSR. The mean values of each functional network were calculated across all participants and across regions within that functional network. Error bars stand for *SEM*. The dashed lines denote the global mean value across all functional networks. A one‐way analysis of variance (ANOVA) was performed on the mean values of each metric within these functional networks. (c) Correlations between regional profiles of weighted NDI across different preprocessing strategies, brain parcellations, and datasets. GSR, global signal regression; NDI, neighbor dispersion index; *SEM*, standard error of the mean

For the weighted networks without a set density threshold, we consistently found that the frontoparietal and salience networks showed higher NDI while primary sensory networks showed lower NDI, and the regional profile of NDI was correlated across preprocessing strategies (without/with GSR, *r* = .71, *p* = 2.4 × 10^–41^), brain parcellations (Power‐264/AAL‐90, *r* = .43, *p* = 1.2 × 10^–4^), and datasets (HCP/FCP, *r* = .54, *p* = 1.4 × 10^–21^). However, the weighted networks showed a narrow dynamic range of NDI across brain regions, particularly without GSR (Figure 9). This result demonstrates that our new metric NDI is applicable to weighted networks.

**Figure 10:**
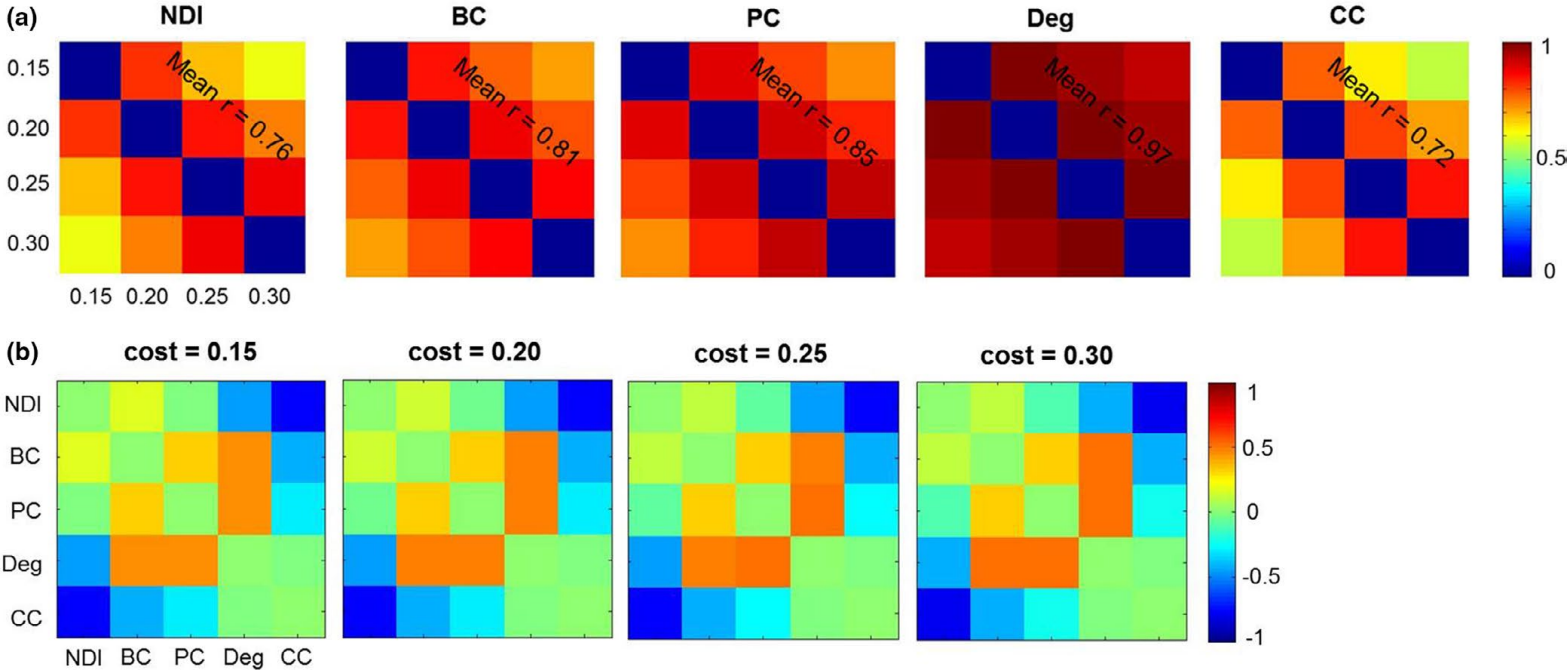
Correlations of each graph metric across different cost thresholds (a), as well as correlations between the graph metrics across different cost thresholds (b). Color bars denote *r* values. BC, betweenness centrality; CC, clustering coefficient; Deg, degree; NDI, neighbor dispersion index; PC, participation coefficient

To test the effect of different network costs, we performed correlation analyses for brain maps of each metric across different cost thresholds. We found that the brain maps are consistent across different cost thresholds for each metric (mean *r* = .76 for NDI; mean *r* = .81 for betweenness centrality; mean *r* = .85 for participant coefficient; mean *r* = .97 for degree; and mean *r* = .72 for clustering coefficient; Figure 9). In addition, we observed the relationships between the graph metrics to be consistent across different cost thresholds (Figure 10). This result suggests that our findings are robust to different network costs.

In addition to the consistency of the regional profile of the NDI, we also found the relationship between the NDI and functional networks to be robust across datasets, network densities, and preprocessing strategies (Figure S5).

### 3.4 Correlations between each graph metric and human intelligence across the whole brain

**Figure 11:**
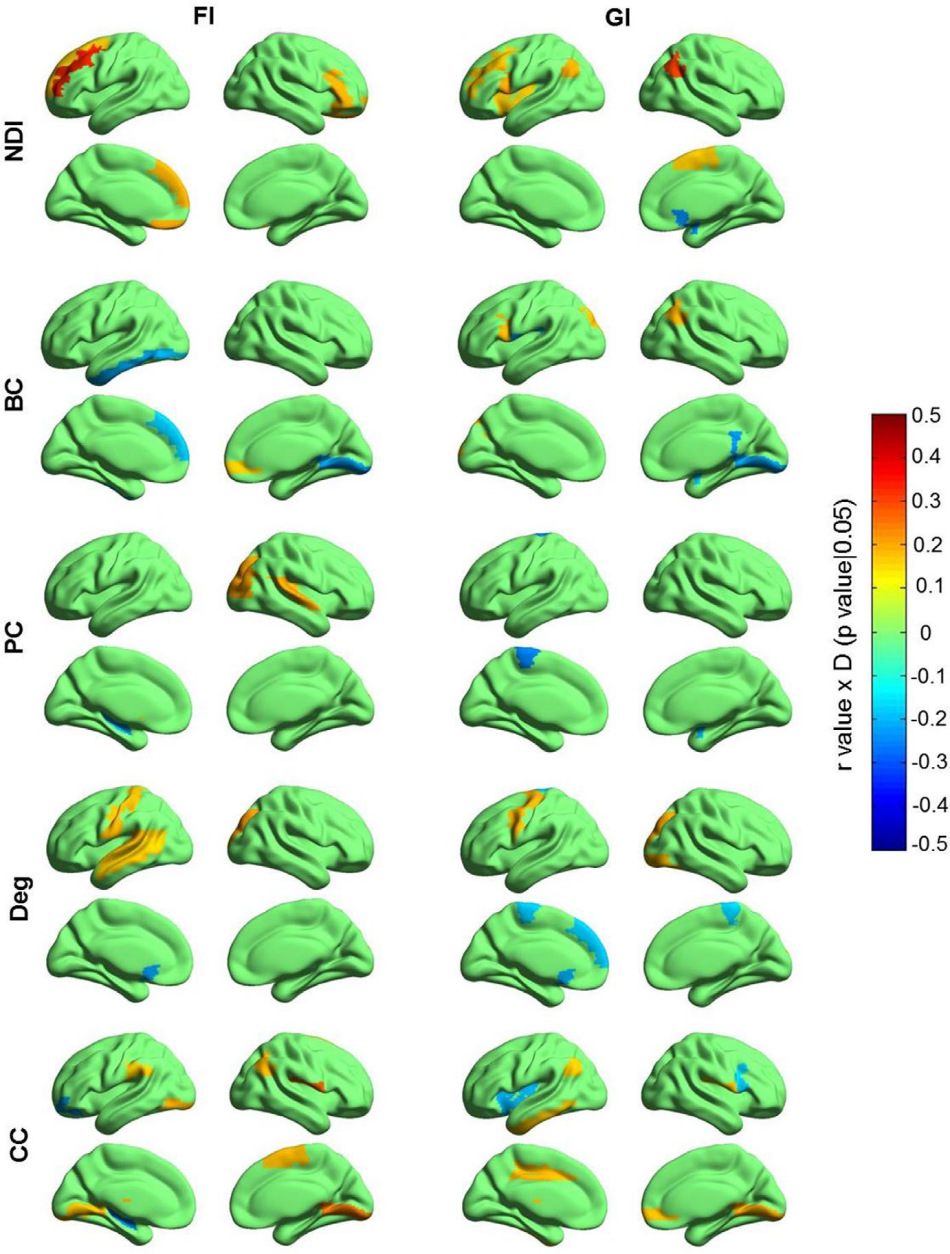
Correlation between each graph metric and human intelligence across whole brain. The color bar denotes *r* value × *D* (*p*‐value|.05), where if *p*‐value < .05, *D* = 1, otherwise *D* = 0. Only correlation between NDI of the left DLPFC and fluid intelligence survives the Bonferroni correction. BC, betweenness centrality; CC, clustering coefficient; Deg, degree; FI, fluid intelligence; GI, general intelligence; NDI, neighbor dispersion index; PC, participation coefficient

To obtain a brain map of regional correlation coefficients, we performed association analysis between each graph metric and human fluid and general intelligence across the whole‐brain regions. We used Penn's Progressive Matrices as a measure of fluid intelligence and estimated general intelligence (including fluid intelligence and crystallized intelligence) by considering scores on multiple measures of cognitive function using factor analysis. For the factor analysis, the Kaiser–Meyer–Olkin test statistic was 0.63 and the statistical significance of Bartlett's test was *p* < .001 (suggesting that it is suitable for factor analysis). In accordance with a previous study (Schultz & Cole, 2016), we treated the first component (explaining 34.1% of the variance) as an evaluation of general intelligence. We generally found that the NDI of prefrontal cortex correlated with fluid intelligence, and the NDI of prefrontal cortex, inferior parietal lobule, and insula correlated with general intelligence, but this was not the case for the other metrics (permutation tests; *p* < .05 uncorrected; Figure 11). After multiple comparison correction was applied, we found that only the NDI of left DLPFC positively correlated with fluid intelligence (permutation test; *p* = .0002; Bonferroni corrected). No significant correlations were observed between any other metrics and human fluid or general intelligence at any brain regions. The raw correlation coefficients are shown in Figure S6. This result suggests that the NDI is different from the other graph metrics in characterizing functional brain organization.

**Figure 12:**
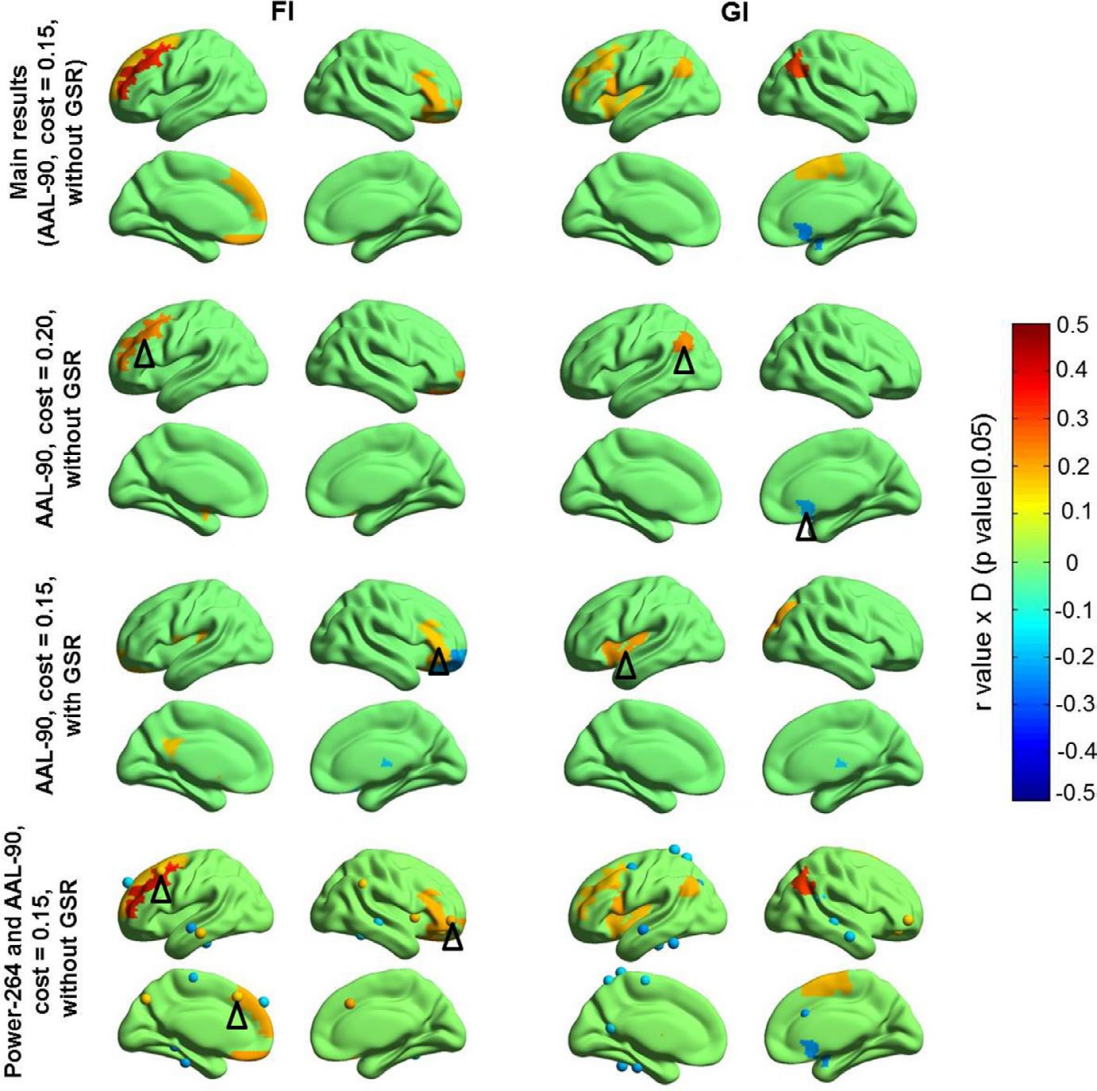
Validation of correlations between NDI and human intelligence across network densities, preprocessing strategies, and brain parcellations. The black triangles indicate the brain regions consistent with those in main result. The color bar denotes *r* value × *D* (*p*‐value|.05), where if *p*‐value < .05, *D* = 1, otherwise *D* = 0. FI, fluid intelligence; GI, general intelligence; GSR, global signal regression; NDI, neighbor dispersion index

To validate the relationship between the NDI and intelligence, we have also calculated the correlations across parameters of preprocessing strategies, network densities, brain parcellations, as well as network types (binary and weighted). We found some consistencies in correlation between NDI and intelligence across these parameters, mainly involving the frontal and parietal cortices, although obvious differences do exist, particularly for the weighted networks (uncorrected *p* < .05; Figures 12 and S7). In addition, we plotted the correlation between fluid intelligence and NDI of the left DLPFC for both AAL‐90 and Power‐264 parcellations, together with a histogram of the null distribution generated during the permutation test (Figure S8).

From the scatter plot of correlation between fluid intelligence and NDI of the left DLPFC, we noticed two subjects who are outliers with lowest NDI equal to 0 (although their fluid intelligence scores are also the lowest). We therefore further examined the functional connectivity patterns of these two subjects as well as two subjects with NDI greater than 0. We found relatively few prefrontal connections in the brain networks of the two outliers, and the left DLPFC has only one neighbor (Figure S9). This result not only suggests that lower fluid intelligence is likely attributed to the relatively lower functional connectivity of prefrontal cortex, but also indicates that NDI could be a good expression of fluid intelligence.

To test whether our main results hold after the removal of these two outliers, we recalculated the correlations between fluid intelligence and the NDI and clustering coefficient of the left DLPFC after excluding the two subjects. We nevertheless found a significant correlation between fluid intelligence and NDI of the left DLPFC (*r* = .18, *p* = .036), although the correlation weakened. In contrast, no significant correlation was found for the clustering coefficient (*r* = −.09, *p* = .18; Figure S10). This result suggests that our main conclusion is not significantly affected by the two outliers.

In addition, a previous study has suggested that subjects with lower overall functional connectivity have more randomly organized brain networks (van den Heuvel et al., 2017). Considering that the connectivity profile of the DLPFC covers almost the whole brain, a more random topology of the network will also influence the NDI of the DLPFC. To test this effect, we first performed correlation analysis between NDI of the DLPFC and overall functional connectivity (i.e., the mean of all positive functional connectivity values). We found significant negative correlation between NDI of the DLPFC and overall functional connectivity both with (*r* = −.25, *p* = .011) and without (*r* = −.20, *p* = .047) the two outliers (Figure S11). This result indicates that lower overall functional connectivity may lead to higher NDI of the DLPFC, which is likely attributed to a more random topology of the network. We subsequently conducted correlation analysis between fluid intelligence and NDI of the DLPFC after regressing out the overall functional connectivity. We consistently observed significant correlations between fluid intelligence and NDI of the DLPFC both with (*r* = .38, *p* = 0) and without (*r* = .25, *p* = .007) the two outliers (Figure S11). Moreover, we found that the correlations were higher after regressing out the overall functional connectivity. This result suggests that our main findings still hold when considering the potential effect of overall functional connectivity.

## 4 DISCUSSION

Although the idea of functional specialization or segregation has led to breakthroughs in cognitive neuroscience, it has also been realized that the specific functional attributes of a brain region are related to its connectivity (integration) with other areas of the brain (Cole et al., 2013; Friston, 2011; Genon et al., 2018; Sallet et al., 2013; Yin et al., 2016). Specifically, a recent study has argued that long‐range connections are key for the functional diversification of brain regions (Betzel & Bassett, 2018). In this study, we present a new graph metric (i.e., NDI), via capturing the topological dissimilarity of immediate neighbors, to characterize the functional diversity of brain regions. Our method emphasizes that the functional role of a brain region is largely determined by the relationship between its neighbors' topology patterns, rather than just its connections (Jbabdi, Sotiropoulos, & Behrens, 2013). A likely reason for this is that the entire topology patterns of its neighbors probably better embody the potential of information spreading from that brain region.

Through comparing NDI with several existing graph metrics commonly used for characterizing nodal capacity of information integration (or network hubs), in hypothetical and empirical functional brain networks, we demonstrated the ability of our proposed graph metric to map the functional diversity of the human brain. Specifically, we found the frontoparietal and salience networks to show higher NDI, while visual, auditory, and sensorimotor networks showed lower NDI, and this was not the case for any other metric. Consistently, a multitask activation study has revealed high functional diversity of frontoparietal and anterior insular regions (Anderson et al., 2013). Based on the structural connectome, a recent study using a method of entropy over the motif participant distribution highlighted high diversity of the cognitive control system (Betzel et al., 2018). Brain regions with higher NDI largely overlap with the multiple‐demand or cognitive control system (Dosenbach et al., 2007; Duncan, 2010), which includes the DLPFC, inferior parietal lobule, dorsomedial prefrontal cortex/dACC, and anterior insula/frontal operculum. The cognitive control system is thought to consist of functionally diverse regions that contribute to flexibly configure information processing in response to changing task demands (Braun et al., 2015; Dosenbach et al., 2007; Posner & Petersen, 1990). In particular, frontoparietal regions play a vital role in flexible top‐down control by biasing information flow across multiple functional systems (Cole et al., 2013; Miller & Cohen, 2001). Nodes of the salience network are capable of detecting and attending to salient goal‐relevant events in a flexible manner, particularly important for the initiation of cognitive control (Chen, Cai, Ryali, Supekar, & Menon, 2016; Ham et al., 2013; Menon & Uddin, 2010). From a network science perspective, our findings not only demonstrate that NDI is capable of describing functional diversity, but also suggest that a high capability in dispersion of information may underlie the adaptive cognitive control of brain regions constituting the frontoparietal and salience networks.

For the association analyses, we observed that human fluid intelligence was associated with the NDI of DLPFC, while no such association for the other metrics was noticed even with an uncorrected *p* < .05. In contrast, the participation coefficient of frontoparietal regions has shown correlation with a wide range of other behaviors (Bertolero et al., 2018). Research in cognitive neuroscience has long sought to understand the nature of individual differences in human intelligence, and different theories have been proposed based on neuroimaging studies (Barbey, 2018). For example, early studies investigating the neural basis of human intelligence primarily implicated the lateral prefrontal cortex (Duncan & Owen, 2000; Duncan et al., 2000; Gray, Chabris, & Braver, 2003). Colom et al argued that human intelligence relates to areas distributed across the brain, not exclusively to frontal lobes (Colom, Jung, & Haier, 2006). Later, emergence of network‐based theories accounted for individual differences in human intelligence on the basis of multiple discrete brain regions, such as parieto‐frontal integration theory (Jung & Haier, 2007) and multiple‐demand theory (Duncan, 2010). Although differences exist across theories, there are important consistencies that the areas associated with human intelligence mainly involve frontal and parietal lobes. In particular, evidence from lesion studies has shown causal correlations between human fluid/general intelligence and lesions in distributed frontal and parietal regions as well as damage to major white matter tracts (Glascher et al., 2010; Woolgar et al., 2010).

Moreover, there is also a wealth of studies exploring relationships between functional connectivity and intelligence (Cole, Yarkoni, Repovs, Anticevic, & Braver, 2012; Ferguson, Anderson, & Spreng, 2017). The original study by van den Heuvel et al (van den Heuvel, Stam, Kahn, & Hulshoff Pol, 2009) reported an association between global network efficiency and intelligence. However, more recent investigation in a larger sample size showed that global efficiency was not associated with intelligence (Kruschwitz, Waller, Daedelow, Walter, & Veer, 2018). Instead, connectivity profiles of the frontoparietal network have been identified to be related to human intelligence (Finn et al., 2015; Hearne et al., 2016). These studies linked network level connectivity/edge to intelligence but did not delve into properties of nodes as our current study did. Notably, for our metric, the connectivity pattern of DLPFC's immediate neighbors is dispersed and almost covers the whole brain. Although the overall functional connectivity can affect global network measure (van den Heuvel et al., 2017), we argue that connectivity topology of the frontoparietal network is likely more sensitive in representing intelligence than a global network measure. Accordingly, we have demonstrated that correlation between fluid intelligence and NDI of the left DLPFC still holds after regressing out the overall functional connectivity. Our finding not only supports previous theories related to human intelligence, but also suggests that NDI is substantially different from other graph metrics in characterizing functional brain organization.

For the validation analysis between NDI and intelligence, we found some consistencies mainly involving the frontal and parietal cortices, although obvious differences were also noticed, especially for the weighted networks. Previous brain network studies have demonstrated that organization principles are robust across spatial scales, but quantitative measures of graph metrics, especially for individual regions, vary substantially (de Reus & van den Heuvel, 2013; Hayasaka & Laurienti, 2010; Wang et al., 2009). In particular, the association between global network efficiency and intelligence has not been successfully replicated across different datasets, even with a wide range of network densities and spatial scales (Kruschwitz et al., 2018; van den Heuvel et al., 2009). Therefore, caution should be noted when interpreting differences in quantitative measures across different preprocessing strategies, network densities, brain parcellations, and datasets, although organization principles of brain network are robust.

A few methodological issues should be considered. First, despite the existence of many centrality metrics (Jalili et al., 2015; Oldham, Fulcher, Parkes, Arnatkeviciute, & Fornito, 2018), to the best of our knowledge, only the participant coefficient (or a similar concept) was recently used and interpreted as a measure of diversity (Bertolero et al., 2017, 2018; Betzel et al., 2018; Schultz et al., 2019). However, the calculation of the participant coefficient requires a decomposition of the functional network into modules, which are highly dependent on the community detection algorithms and may result in different modules on every run (Betzel et al., 2018; Guimera & Nunes Amaral, 2005; Power et al., 2013; Sporns & Betzel, 2016). In addition, Bertolero and colleagues recently defined a “diverse club,” containing nodes with both a high participant coefficient and a high degree, to describe integrative network function (Bertolero et al., 2017). Both diverse club and classic rich club (van den Heuvel & Sporns, 2013a) are identified based on predefined network hubs (i.e., nodes with higher degree). In contrast to our finding that frontoparietal and salience networks showed higher diversity, their result showed that nodes from the diverse club were mainly involved in ventral attention network and salience network, and nodes from rich club were primarily included in visual and dorsal attention networks. This suggests that our NDI captures distinct topological properties from diverse club and rich club. Moreover, our proposed measure is relatively simple, and its calculation is not based on other graph metrics such as modularity and degree.

Second, there was a negative correlation between NDI and clustering coefficient. Mathematically, NDI may capture some common information with the clustering coefficient (i.e., the NDI captures entire topological patterns of immediate neighbors of a node, while the clustering coefficient captures only direct connections among immediate neighbors of a node). However, the correlation was not perfect, which means that NDI cannot be well described by the clustering coefficient through a linear relation. More importantly, we found that correlations of NDI and clustering coefficient with human intelligence were distinct across the whole brain. This suggests specificity of NDI in capturing information from network topology compared to clustering coefficient.

Third, although there were brain‐wide correlations for the graph metrics between without and with GSR, the specific brain regions or functional networks were found to be influenced by GSR. For example, NDI of default mode network relatively decreased and subcortical network increased after performing GSR (i.e., the NDI of default mode network is significantly higher (*p* < .05 with Bonferroni correction) than that of subcortical network without GSR. Inversely, the NDI of subcortical network is significantly higher (*p* < .05 with Bonferroni correction) than that of default mode network after performing GSR), and participant coefficient of subcortical network relatively increased with GSR. Consistently, previous studies also reported that both global and local graph theoretical measures were impacted by GSR (Liang et al., 2012; Sinclair et al., 2015). However, including GSR in the preprocessing of resting‐state functional connectivity data is not inherently right or wrong because of no accepted gold standard (Murphy & Fox, 2017). Furthermore, we found that degree and clustering coefficient were more robust to the GSR compared with NDI and participant coefficient. It is possible that NDI and participant coefficient are more sensitive to the changes of network topology.

Finally, although our new metric NDI is applicable for weighted networks, connectivity weights seem to play a minor role in sparse weighted networks. The sparser the weighted networks, the narrower the distribution of connectivity weights (i.e., the difference of connectivity weights is small). Perhaps, this is why connectivity weights appear to play a minor role in sparse weighted networks. It is possible that connectivity topology plays a more crucial role in the NDI of nodes than connectivity weights do, especially in sparse weighted networks. Moreover, we found that weighted networks without a set density threshold showed a narrow dynamic range of NDI across brain regions, particularly without GSR. Previous evidence suggests that GSR may introduce negative correlations (Murphy & Fox, 2017). It is likely that weighted networks without GSR could be denser than those with GSR (negative correlations were set to zero). In addition, we did not find a significant correlation between NDI and intelligence in frontoparietal regions for weighted networks. This suggests that NDI might not be sensitive in distinguishing the roles of different nodes in dense weighted networks.

In conclusion, this study proposes a novel graph measure to describe the functional diversity of brain regions on the basis of topological dissimilarity between immediate neighbors. Our method successfully demonstrated that brain regions showing higher functional diversity are primarily located in the frontoparietal and salience networks. In contrast, unimodal sensory regions show lower functional diversity. Furthermore, the NDI of DLPFC is associated with variations in human fluid intelligence, while no such association for the other metrics commonly used for characterizing network hubs was noticed even with an uncorrected *p* < .05. Our findings not only provide new insight into the network architecture of brain function, but also shed light on individual differences in human intelligence. Moreover, this new graph method has potential for exploring how functional diversity of brain regions evolves during brain development or is disrupted in neuropsychiatric disorders.

## CONFLICT OF INTEREST

The authors declare no competing financial interests.

### DATA ACCESSIBILITY

The data that support the findings of this study are openly available in ConnectomDB at https://www.humanconnectome.org/study/hcp-young-adult and NITRC at http://fcon_1000.projects.nitrc.org/fcpClassic/FcpTable.html.
