# Supplementary material for: A graph representation of functional diversity of brain regions
Source: Brain Behav. 2019 Jul 27;9(9):e01358. doi: 10.1002/brb3.1358 (PMC6749480; doi:10.1002/brb3.1358)
Supplement: Supplementary file 1 [file BRB3-9-e01358-s001.doc]

**Supplementary Information**

**Title:** A Graph Representation of Functional Diversity of Brain Regions

**Authors:** Dazhi Yin, Xiaoyu Chen, Kristina Zeljic, Yafeng Zhan, Xiangyu Shen, Gang Yan, and Zheng Wang

**Supplementary Methods**

**Calculation of Small-Worldness**

We employed the commonly used clustering coefficient and shortest path length to quantify the small-world property of functional brain networks. The absolute clustering coefficient *C_i_* of a node *i* is defined as the ratio of the number of existing edges between neighbors of *i*, and the maximum possible number of edges between neighbors of *i*:

$C_{i}=\frac{E_{i}}{K_{i}\left(K_{i}-1\right)/2}$,

where *E_i_* is the number of edges between the neighbors of *i*, and *K_i_* is the degree of *i*. The absolute clustering coefficient of a network is the average of the absolute clustering coefficient of all nodes:

$C_{p}=\frac{1}{N}\sum_{i\in G} C_{i}$,

*C_p_* is a measure of the extent of cliquishness of the network. To deal with disconnected nodes, we calculated the absolute shortest path length of a network using harmonic mean of distance :

$\frac{1}{L_{p}}=\frac{1}{N\left(N-1\right)}\sum_{i\neq j\in G} \frac{1}{d_{ij}}$,

*L_p_* is a measure of efficiency of the network.

Practically, a network can be categorized as a small-world network if it has similar absolute path lengths but a higher absolute clustering coefficient than random networks, that is$\gamma=C_{p}^{real}/C_{p}^{rand}>1$, and $\lambda=L_{p}^{real}/L_{p}^{rand}\approx1$.$C_{p}^{rand}$and $L_{p}^{rand}$denote clustering coefficients and path lengths of random networks. These two conditions can also be summarized into a scalar quantitative measurement, small-worldness$\sigma=\gamma/\lambda$, which is typically >1 for small-world networks . For comparison purposes, we generated random networks using the random rewiring procedure that preserves the degree distribution of the real network .

**
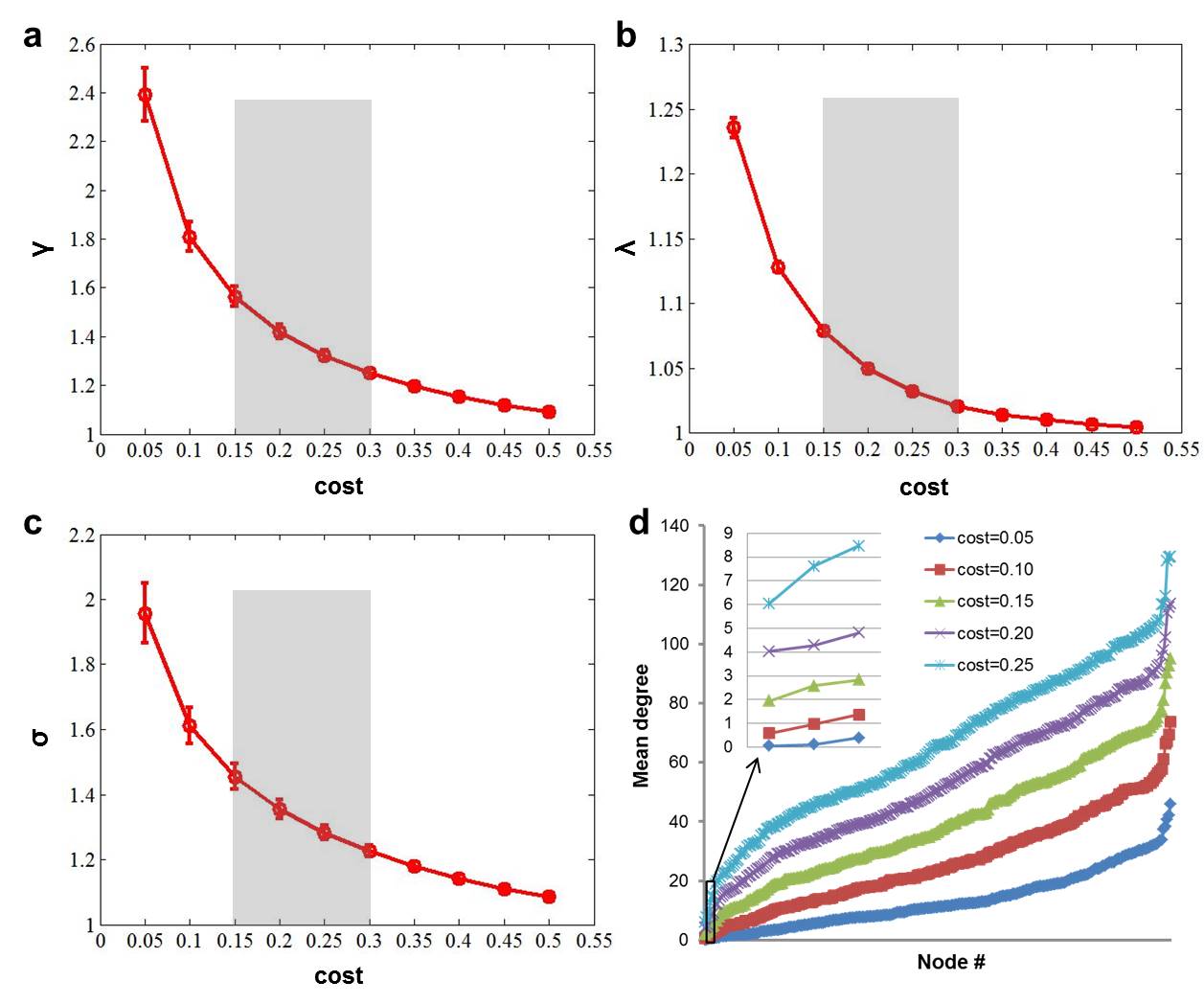
**

**Figure S1:** Selection of network cost thresholds. Normalized clustering coefficient (a), normalized shortest path length (b), small-worldness (c), and nodal mean degree (d) of the functional brain networks were calculated across a wide range of cost (0.05≤cost≤0.5, with an incremental interval of 0.05). Here, the functional brain networks were constructed from the HCP dataset based on Power-264 parcellation. The shadow area indicates economic and small-world regime. Nodal mean degree is shown in ascending order. The error bars denote SEM. Threshold cost = 0.15 was selected by simultaneously considering economic and small-world properties of the brain network. HCP, human connectome project and SEM, standard error of the mean.


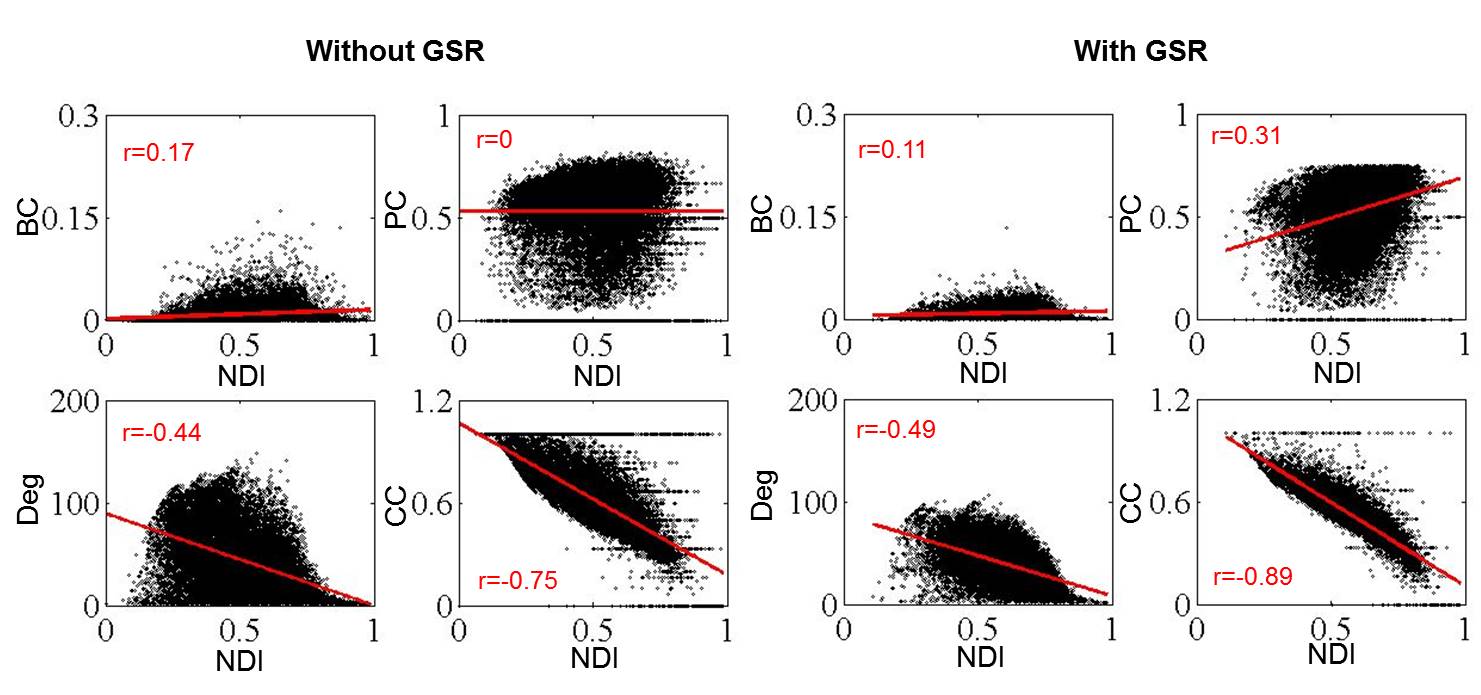


**Figure S2:** To identify distinguishability of NDI, Pearson correlation analyses between NDI and the other metrics were performed across the whole brain both without and with GSR, and r values are reported. NDI, neighbor dispersion index; BC, betweenness centrality; PC, participation coefficient; Deg, degree; CC, clustering coefficient; and GSR, global signal regression.


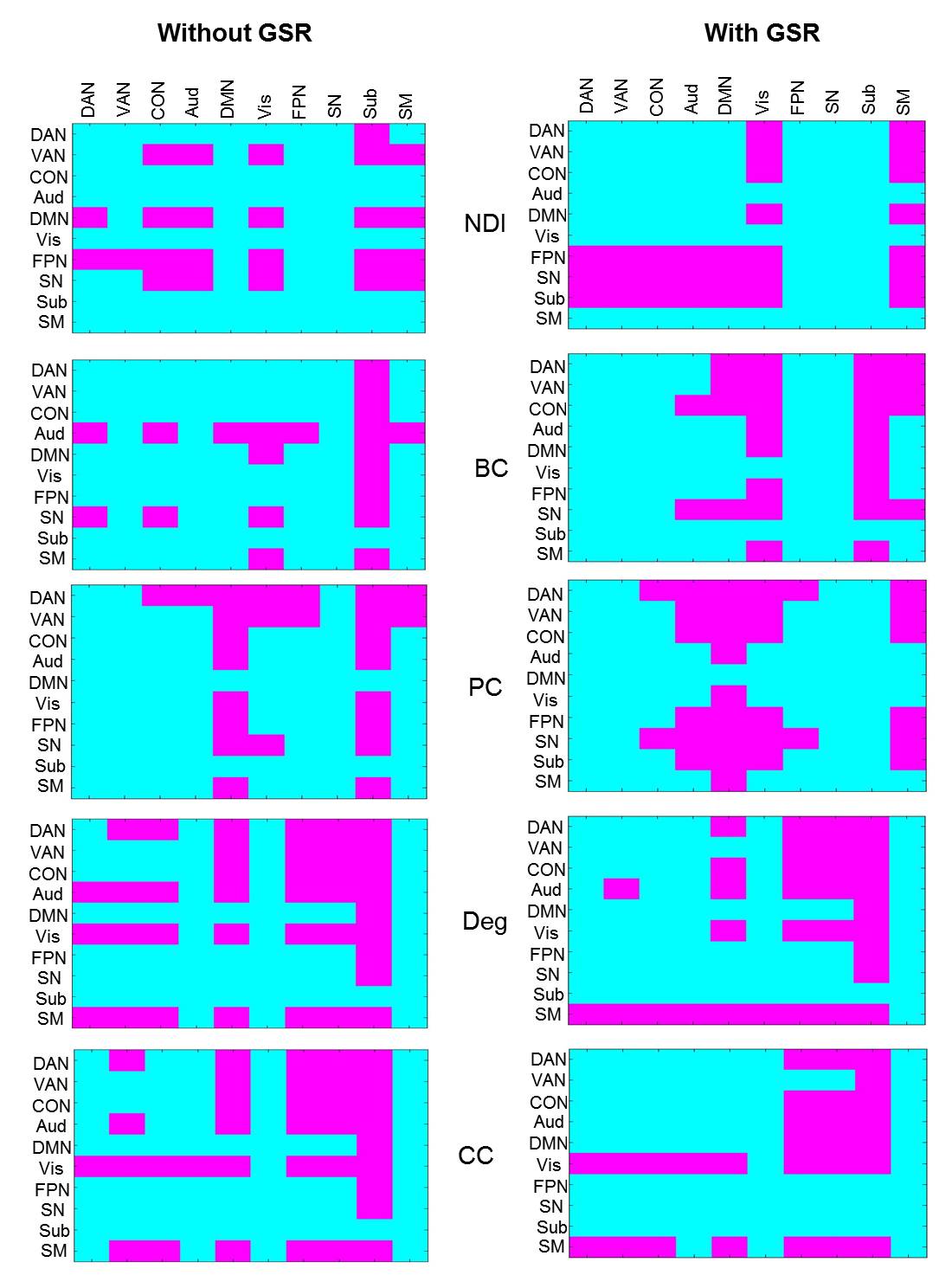


**Figure S3:** Post hoc two-sample t-tests were conducted to assess the significant differences in mean values of each metric between any two functional networks. The threshold of p < 0.05 with Bonferroni correction was considered statistically significant. Each element *a_ij_* of the matrix denotes the mean value of the functional network *i* significantly greater (pink) than the functional network *j*. NDI, neighbor dispersion index; BC, betweenness centrality; PC, participation coefficient; Deg, degree; CC, clustering coefficient; DAN, dorsal attention network; VAN, ventral attention network; CON, cingulate-opercular network; Aud, auditory network; DMN, default mode network; Vis, visual network; FPN, frontoparietal network; SN, salience network; Sub, subcortical network; SM, sensorimotor network; SEM, standard error of the mean; and GSR, global signal regression.


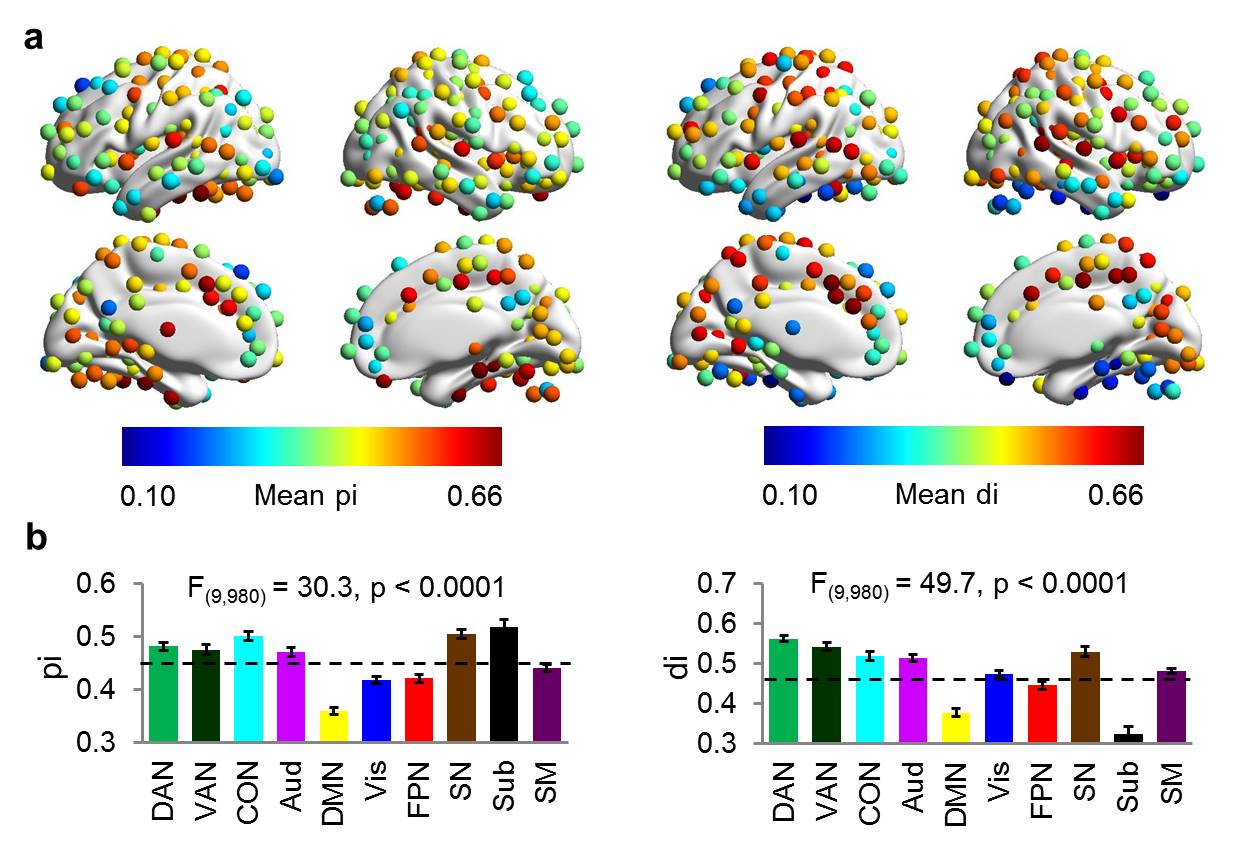


**Figure S4:** (a) Mean pi and di values of each node across participants. The color bar denotes the magnitude of mean values. (b) Mean pi and di values of each functional network. The mean values of each functional network were calculated across all participants and across regions within that functional network. Error bars stand for SEM. The dashed lines denote the global mean value across all functional networks. A one-way analysis of variance (ANOVA) was performed on the mean values of each metric within these functional networks. pi = participation index; di = dispersion index; DAN, dorsal attention network; VAN, ventral attention network; CON, cingulate-opercular network; Aud, auditory network; DMN, default mode network; Vis, visual network; FPN, frontoparietal network; SN, salience network; Sub, subcortical network; SM, sensorimotor network; and SEM, standard error of the mean.


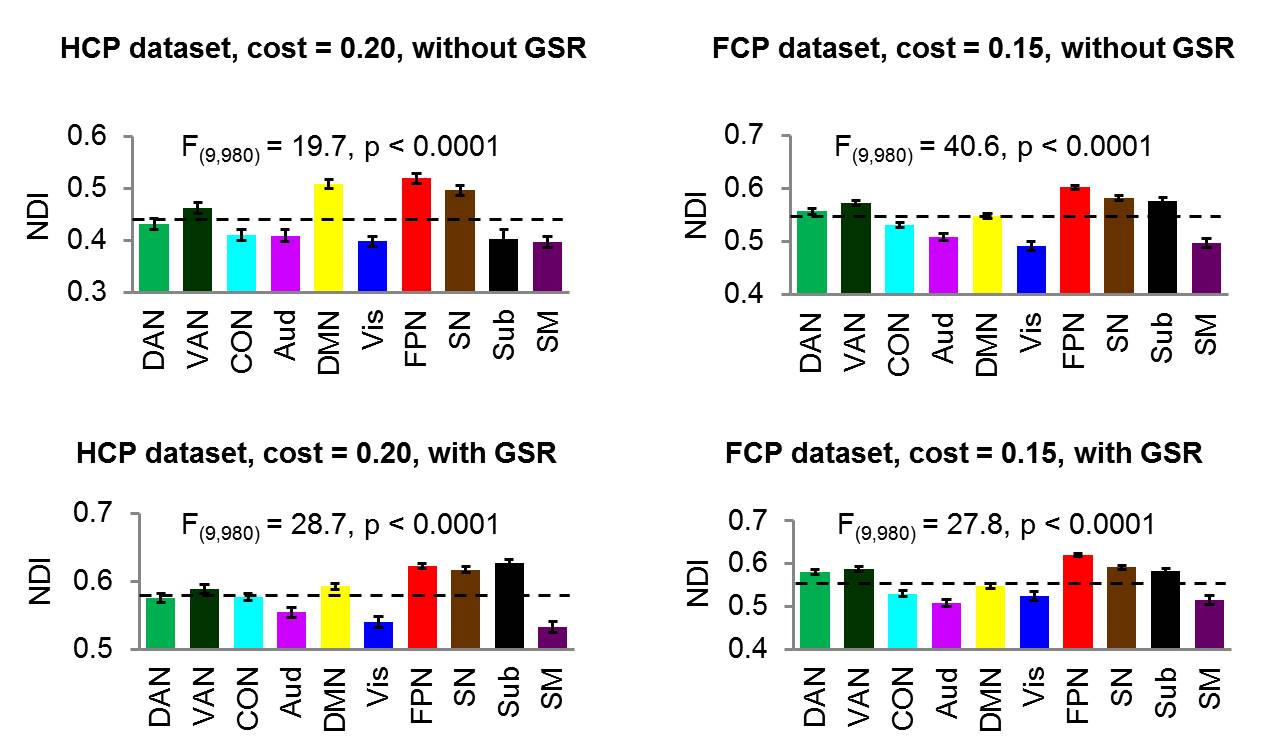


**Figure S5:** The relation between the NDI and functional networks is robust across datasets, network densities, and preprocessing strategies. NDI, neighbor dispersion index; HCP, human connectome project; FCP, functional connectomes project; GSR, global signal regression; DAN, dorsal attention network; VAN, ventral attention network; CON, cingulate-opercular network; Aud, auditory network; DMN, default mode network; Vis, visual network; FPN, frontoparietal network; SN, salience network; Sub, subcortical network; and SM, sensorimotor network.


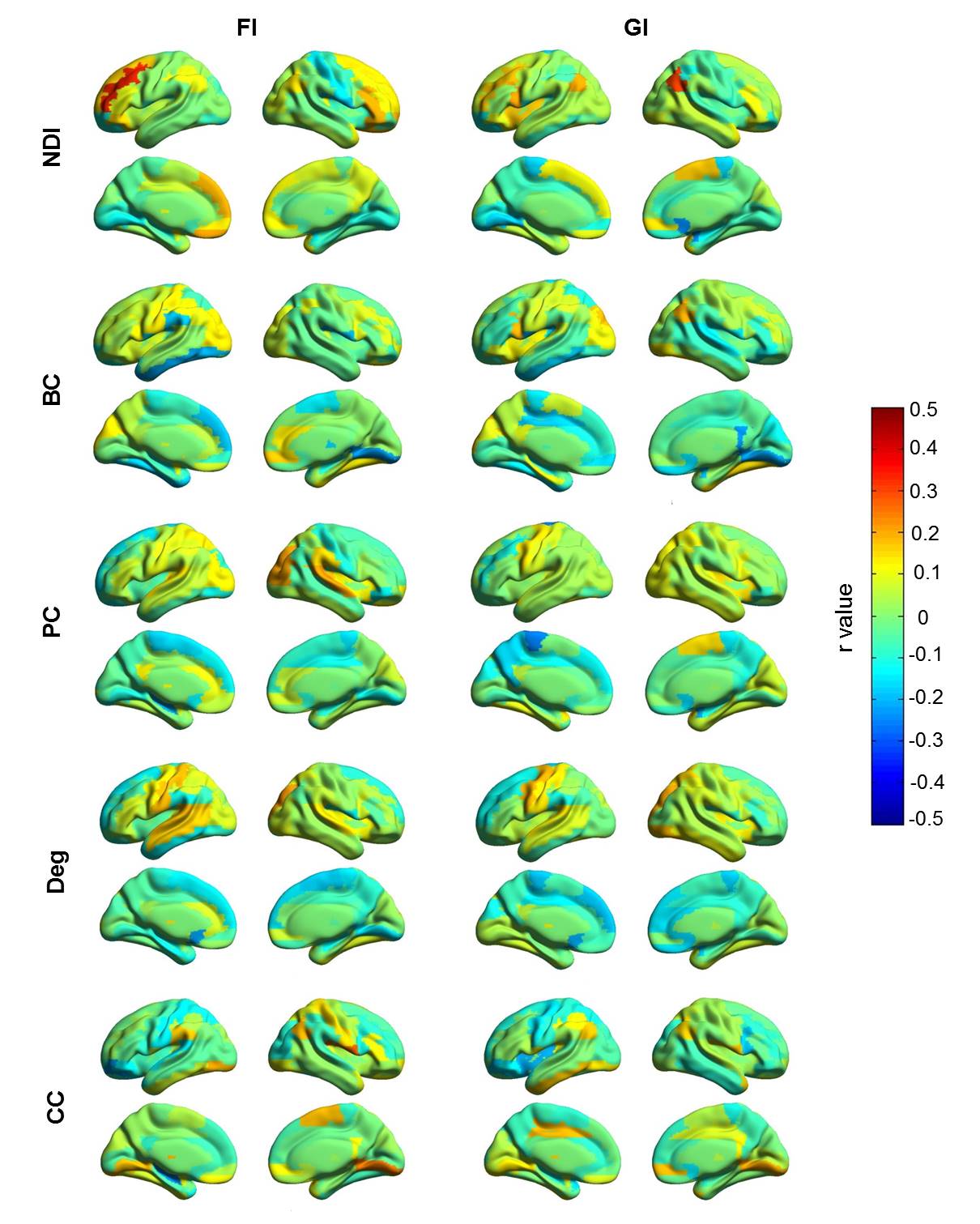


**Figure S6:** Brain map of raw correlation coefficients between each graph metric and human intelligence. The color bar denotes the magnitude of r values. FI, fluid intelligence; GI, general intelligence; NDI, neighbor dispersion index; BC, betweenness centrality; PC, participation coefficient; Deg, degree; and CC, clustering coefficient.


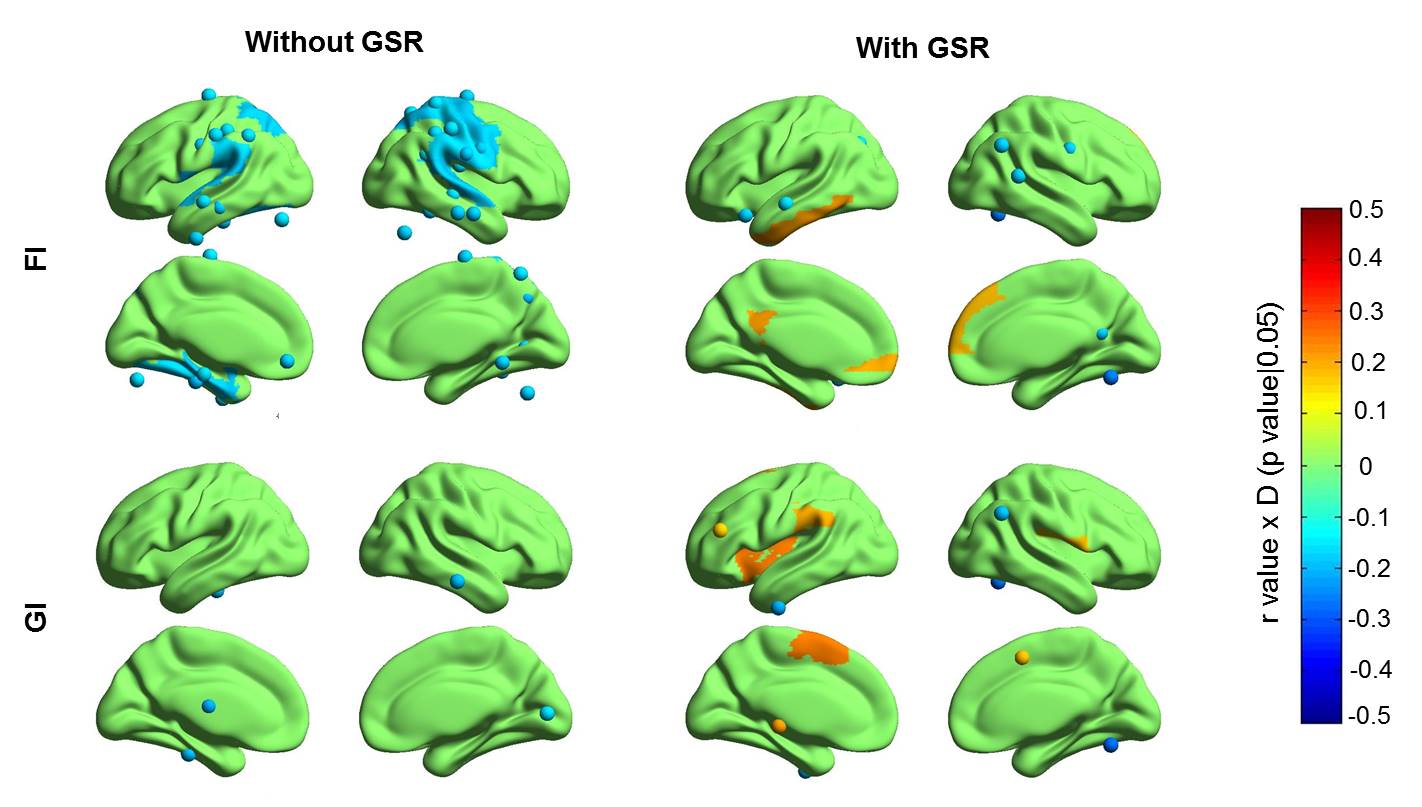


**Figure S7:** Overlapped correlation map (based on AAL-90 and Power-264 parcellations) between NDI and human intelligence for weighted networks without setting a density threshold. The color bar denotes r value x D (p value|0.05), where if p value < 0.05, D = 1, otherwise D = 0. FI, fluid intelligence; GI, general intelligence; NDI, neighbor dispersion index; and GSR, global signal regression.


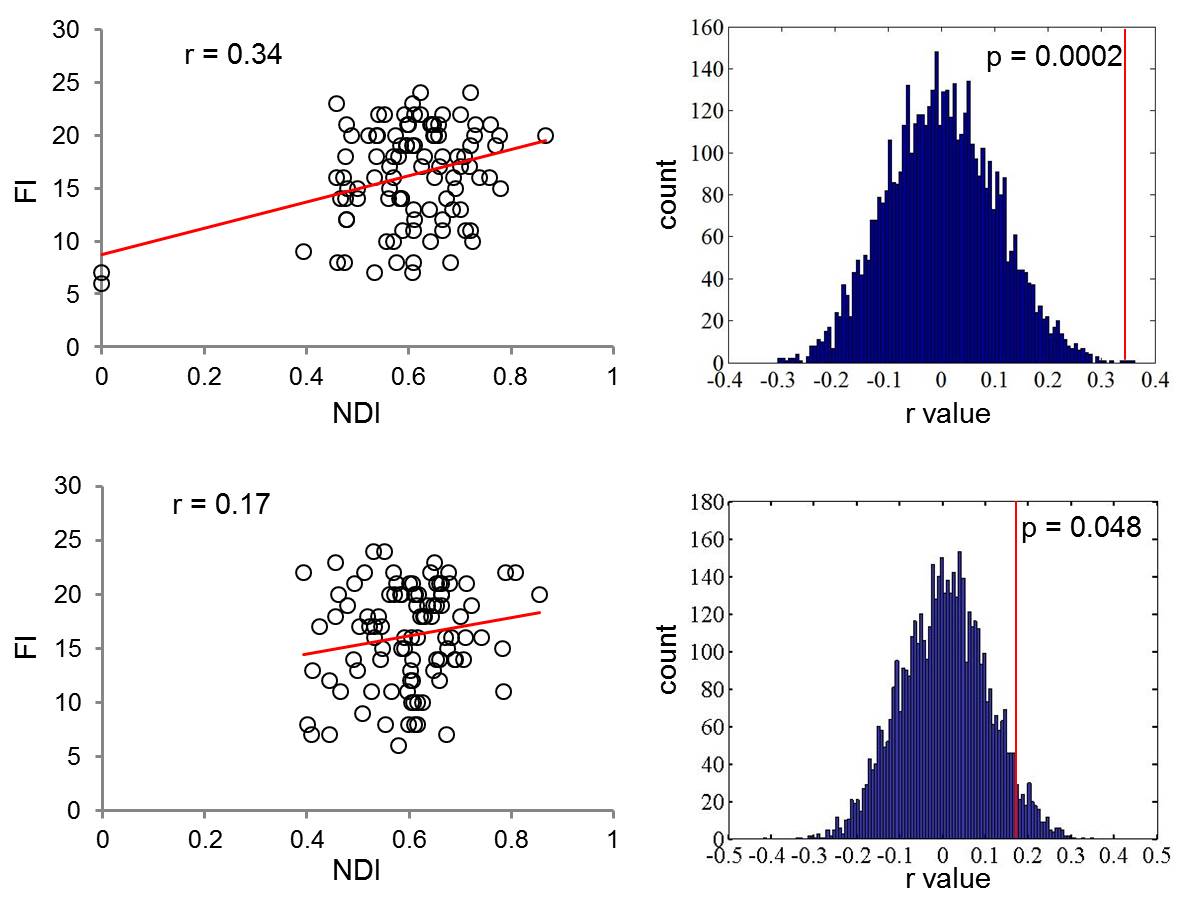


**Figure S8:** Correlation between FI and NDI of the left DLPFC for AAL-90 (top row) and Power-264 parcellations (bottom row), together with a histogram of the null distribution generated during the permutation test. FI, fluid intelligence; NDI, neighbor dispersion index; and DLPFC, dorsolateral prefrontal cortex.


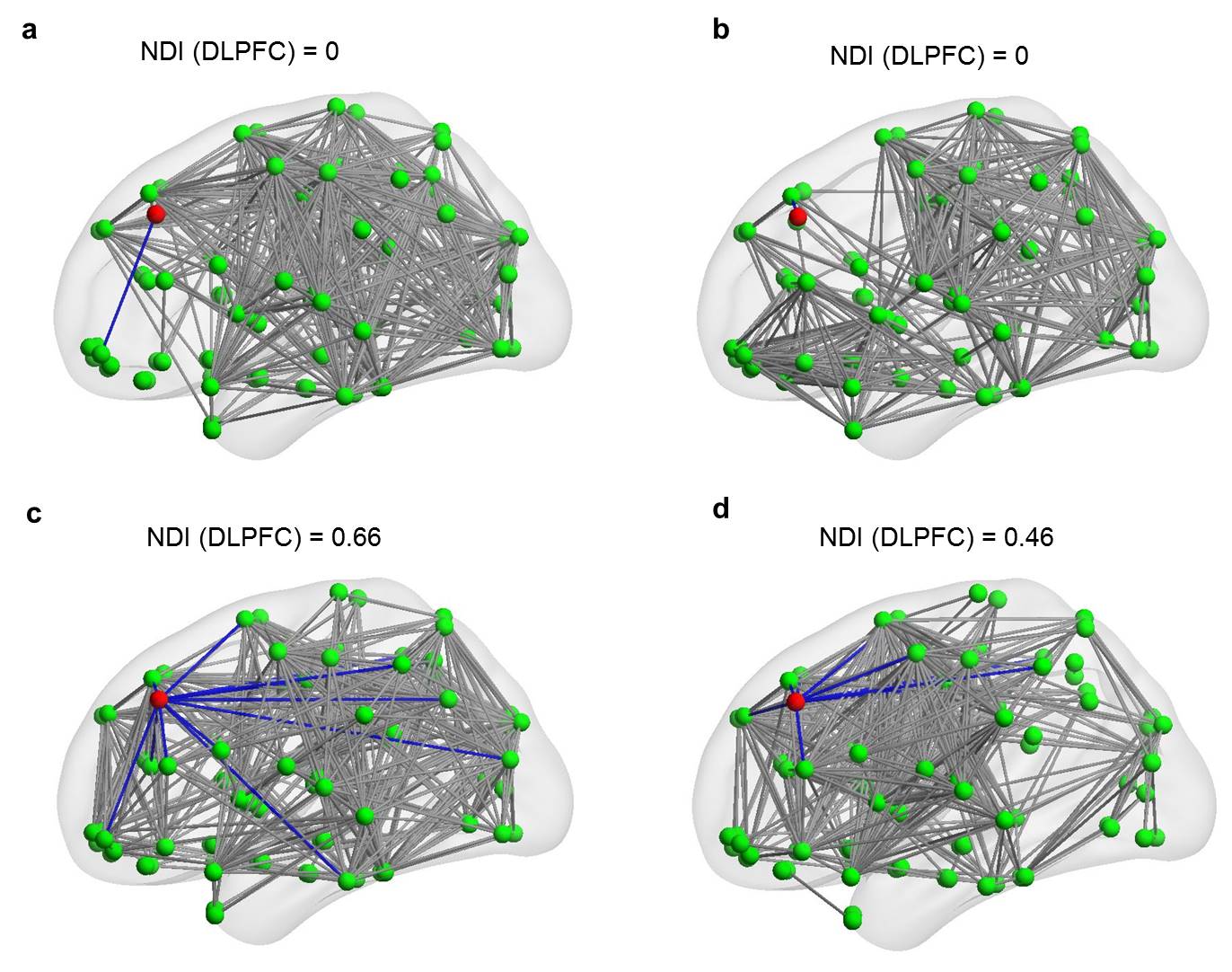


**Figure S9:** The functional connectivity patterns for the two subjects (HCP#100408, HCP#189450) with NDI of the left DLPFC equal to 0 (a, b) as well as two subjects (HCP#100307, HCP#110411) with NDI of the left DLPFC greater than 0 (c, d) are shown. The threshold of network density is 0.15, which means the strongest 15% of functional connections were reserved. The NDI of the left DLPFC equal to 0 observed in these two subjects is attributed to the DLPFC having only one neighbor. The balls denote brain regions of the AAL template and the lines denote functional connections between brain regions. Therein, the red ball indicates left DLPFC and blue lines indicate its connections. NDI, neighbor dispersion index; CC, clustering coefficient; and DLPFC, dorsolateral prefrontal cortex.


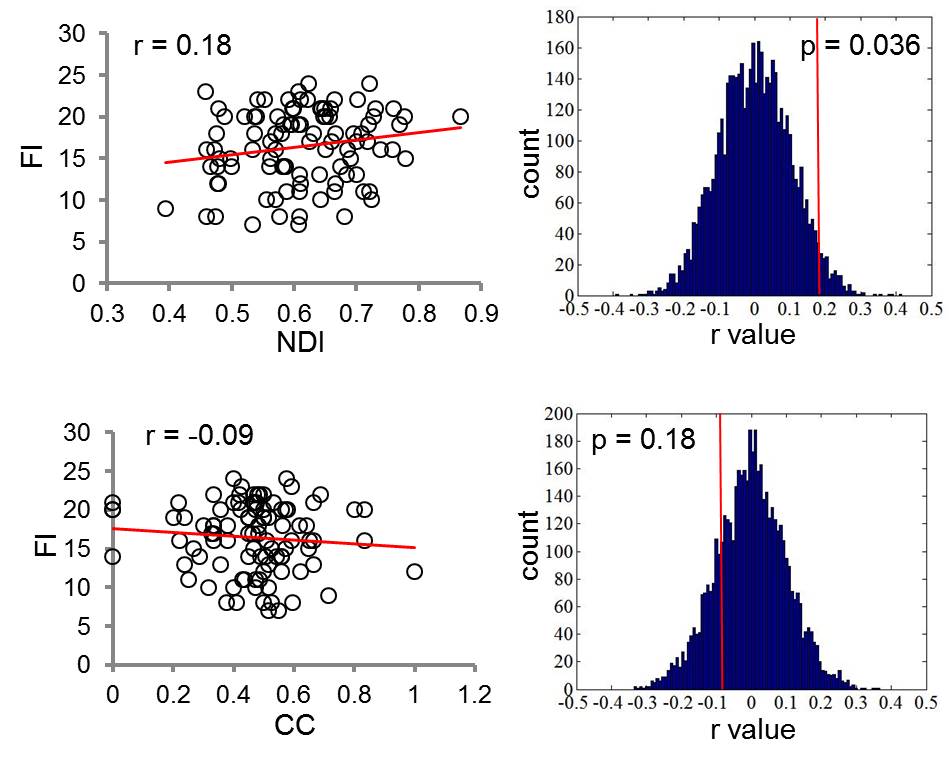


**Figure S10:** Correlation between FI and NDI (top row) and CC (bottom row) of the left DLPFC, together with a histogram of the null distribution generated during the permutation test. FI, fluid intelligence; NDI, neighbor dispersion index; CC, clustering coefficient; and DLPFC, dorsolateral prefrontal cortex.


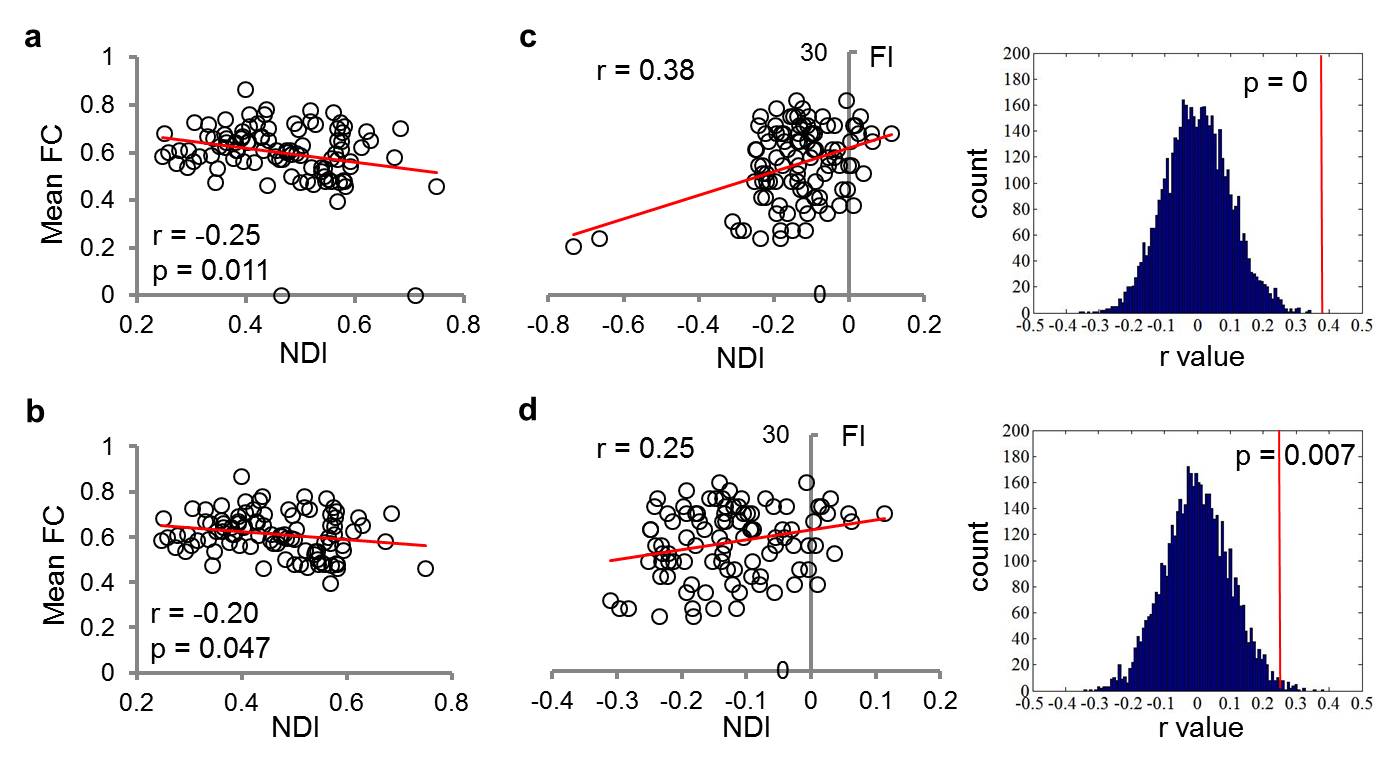


**Figure S11:** Correlation between NDI of the left DLPFC and mean FC is exhibited both with (a) and without (b) the two outliers. Correlation between FI and NDI of the left DLPFC after regressing out the overall functional connectivity, together with a histogram of the null distribution generated during the permutation test, are shown both with (c) and without (d) the two outliers. FC, functional connectivity; FI, fluid intelligence; NDI, neighbor dispersion index; and DLPFC, dorsolateral prefrontal cortex.

**References:**

Achard, S., Salvador, R., Whitcher, B., Suckling, J., & Bullmore, E. (2006). A resilient, low-frequency, small-world human brain functional network with highly connected association cortical hubs. *The Journal of neuroscience : the official journal of the Society for Neuroscience, 26*(1), 63-72.

Bullmore, E. T., & Bassett, D. S. (2011). Brain graphs: graphical models of the human brain connectome. *Annual Review of Clinical Psychology, 7*, 113-140.

Liu, Y., Liang, M., Zhou, Y., He, Y., Hao, Y., Song, M., . . . Jiang, T. (2008). Disrupted small-world networks in schizophrenia. *Brain : a journal of neurology, 131*(Pt 4), 945-961.

Maslov, S., & Sneppen, K. (2002). Specificity and stability in topology of protein networks. *Science, 296*(5569), 910-913.

Newman, M. E. J. (2003). The structure and function of complex networks. *Siam Rev, 45*(2), 167-256.

Watts, D. J., & Strogatz, S. H. (1998). Collective dynamics of 'small-world' networks. *Nature, 393*(6684), 440-442.
